# Probiotic characteristics and whole genome sequencing of *Pediococcus pentosaceus* SNF15 and its protective effect on mice diarrhea induced by *Escherichia coli* K99

**DOI:** 10.3389/fvets.2025.1524658

**Published:** 2025-03-13

**Authors:** Yalan Su, Mingque Feng, Jingdi Tong, Xiangfu Wen, Meiyi Ren, Deyuan Song, Jinshang Song, Xiaohan Li, Qinna Xie, Jia Cheng, Mingchao Liu

**Affiliations:** College of Veterinary Medicine, Hebei Agricultural University, Baoding, China

**Keywords:** *Pediococcus pentosaceus* SNF15, *Escherichia coli*, diarrhea, probiotic properties, whole genome sequencing, gut microbiota

## Abstract

*Escherichia coli* (*E. col* iK99) is one of the primary pathogens that cause infectious calf diarrhea, resulting in mortality and causing economic losses. Probiotics have been widely researched for their positive impact on inhibiting the growth of pathogenic bacteria and enhancing immunity and gut health as alternatives to antibiotics. This study isolated one probiotic from healthy calf feces: *Pediococcus pentosaceus* SNF15 (*P. pentosaceus* SNF15). In vitro assessments included growth character and acid-producing ability, bile salt and artificial gastroenteric fluid tolerance, Caco-2 adhesion, hemolysis screening, and antibiotic susceptibility. Whole-genome sequencing identified immunomodulatory, antimicrobial, and metabolic genes. A murine model evaluated probiotic efficacy against *E. coli* K99, outcomes included clinical indices (fecal score, weight), histopathology (H&E), inflammatarty factor (qRT-PCR and ELISA), tight junction proteins and mucin (immunohistochemistry detection). Finally, 16S rRNA sequencing was performed to compare the composition and relative abundance of the gut microbiota among the different groups. *P. pentosaceus* SNF15 demonstrated excellent growth performance and acid production capacity, bile salt and artificial gastroenteric fluid resistance, Caco-2 cells adhesion and safety (γ-hemolysis, antibiotic sensitivity) Genomic analysis revealed to immune, anti-inflammatory, antagonistic pathogens, and carbohydrate utilization, including secondary bile acid, nicotinate and nicotinamide. The animal tests showed that the *P. pentosaceus* SNF15 treatment protects against *E. coli* K99 infection, as evidenced by clinical symptoms, including weight loss, fecal score, liver atrophy, and spleen enlargement occurred histological damage. Compared with the CN group, the supplementation of *P. pentosaceus* SNF15 strains ameliorated the damage of jejunum and the content of tight junction proteins occludin, claudin, ZO-1, and MUC2 and decreased the levels of IL-6, IL-1β, and TNF-α in jejunum. The 16S rDNA sequence results showed that infection with Escherichia coli K99 led to an imbalance in gut microbiota; the proportion of *Firmicutes* and *Bacteroidetes* decreased, and *Proteobacteria* increased. *P. pentosaceus* SNF15 helps improve intestinal microbial composition and prevents this trend. *P. pentosaceus* SNF15 supplementation can prevent and treat the clinical symptoms, intestinal epithelial mucosal integrity, intestinal permeability, and immune-related cytokines and regulate the intestinal microbiota in E. coli K99-infected mice. This research revealed that *P. pentosaceus* SNF15 possesses desirable probiotic characteristics and could be used as a potential probiotic to remit neonatal calf diarrhea, caused by *E. coli* K99 infection.

## 1 Introduction

Neonatal calf diarrhea (NCD) is a symptom of thin, watery stools and hypothermia. This condition is commonly seen in dairy farms (1). NCD is still a significant cause of productivity and economic loss to cattle farms worldwide (2). *Escherichia coli* (*E. coli*) is one of the major pathogenic microorganisms that cause NCD. At Ningxia, China, the detection rate of *E. coli* K99 in NCD is 20.00% (3). In the US, the diarrhea morbidity of preweaned heifer calves is 20%, and extensive antibiotic use is common in the therapeutic process (4).

Now, antibiotics are always used when treating NCD in practical applications. Overuse of antibiotics leads to the emergence of multi-drug-resistant pathogens (5) and food residues (6) and other problems. These have become serious global public health issues. Therefore, finding a safe and effective substitute for antibiotics is urgent. Various research has indicated that the health promotion effects of probiotics (7), essential oils (8), Chinese medicines (9), and fatty acids (10), including reducing the incidence of NCD, regulating gut microbial diversity, immunomodulation, and enhancing calf growth; some of them can even directly antagonize the growth of pathogens.

Many studies have indicated that probiotics can treat various diseases, suppress pathogens, and improve immunity, thus being able to be used as antibiotic alternatives (10). Currently, dairy cattle farms generally use probiotics to increase the efficiency of calf rearing and prevent and treat various diseases (11). Calves taking *L. acidophilus*, *B. subtilis*, and *S. cerevisiae* orally can effectively improve the gut barrier damage caused by infectious *E. coli* K99, including expression of IL-1β, TNF-α, and NF-κB in the gut barrier, and enhance intestinal microbiota diversity (12). *Limosilactobacillus reuteri* RGW1 isolated from feces of the healthy calf can elevate anti-inflammatory agent IL-10 levels, while cell-free supernatant of RGW1 decreases the level of proinflammatory cytokines TNF-a levels in the serum (7).

This study isolated and identified a new *Pediococcus pentosaceus* SNF15 strain originating from healthy calf excrement. Assess potential probiotic traits of *Pediococcus pentosaceus* SNF15, including antibacterial activity, analysis of bacteriostatic substances, growth and acid production characteristics, survival ability *in vitro* under artificial gastroenteric fluid and bile, adherence ability to Caco-2 cells, hemolysis, and antibiotic sensitivity. To further study the mode of action of *Pediococcus pentosaceus* SNF15, whole genome sequencing of *Pediococcus pentosaceus* SNF15 was conducted. The essential genome functions of the GO, eggNOG, KEGG, and other databases were annotated to provide a bioinformatics basis for isolated strain probiotic properties. We assessed the impact of supplementing probiotics *Pediococcus pentosaceus* SNF15 on *E. coli* K99-infected mice, immunological indices, jejunum morphology and barrier function, and cecum intestinal flora. This research provides a theoretical basis for the prevention and treatment of NCD.

## 2 Materials and methods

### 2.1 Isolation, culture, and safety identification of probiotics

Ten copies of 2–14 days of healthy calves’ fresh feces samples were collected from a dairy cattle farm (Ding Zhou, Hebei, China). The feces were fully homogenized, spread on MRS agar (Qingdao hopebiol, China), and hermetically cultured at 37°C without O_2_ for 24 h, MRS plate was sealed with a sealing membrane. White, pinpointed single colonies were selected for purification according to Berger’s manual for bacterial identification (13). The purified bacteria were inoculated in MRS broth and static cultured at 37°C without O_2_ for 24 h, and the centrifugal tube is sealed with a sealing membrane to isolate oxygen. DNA was extracted using the TIANamp Bacteria DNA Kit (TIANGEN BIOTECH (BEIJING) CO., LTD., China). PCR amplification was performed using bacterial 16S rRNA universal primers: 27F (5′-AGAGTTGATCCTGGCTCAG-3′) and 1492R (5′-TACGGCTACCTTGTACGACTT-3′). The PCR product was detected by 1% agar gel electrophoresis. Then, bands were observed and analyzed using a gel imaging system. The banded PCR products were sent to Shanghai Sangon Biological Engineering Technology & Services Co for sequencing. Using the nucleotide BLAST tool, the sequenced PCR products were compared to those in the National Center for Biotechnology Information (NCBI) database.

#### 2.1.1 Hemolytic activity

The hemolytic activity of *P. pentosaceus* SNF15 was carried out as described by Rastogi et al. (14). Inoculate *P. pentosaceus* SNF15 activated on MRS agar into TSA (Qingdao hopebiol, China) blood agar containing 5% (v/v) off fiber sheep blood (Qingdao hopebiol, China) and cultured for 24 h at 37°C. Plates were then observed for hemolysis for β-hemolysis (clear halo around each colony), α-hemolysis (greenish halos around each colony), and γ-hemolysis (no halo around each colony). *Staphylococcus aureus* (CVCC186158, Guangzhou Legend Biotech Co., LTD) showing β-hemolysis was used as the positive control.

#### 2.1.2 Antibiotic susceptibility testing

Antibiotic susceptibility testing of the isolated strain was determined by agar disk diffusion assay on MRS agar (Qingdao hopebiol, China), the results indicated as susceptible, S (inhibition zone ≥21 mm); intermediate, I (15 < inhibition zone <21 mm) and resistance R (inhibition zone ≤15 mm) according to Yang et al. (15). *P. pentosaceus* SNF15 was inoculated in MRS broth (Qingdao hopebiol, China) and cultured for 24 h at 37°C; the concentration of the isolated strain solution was adjusted to 1.5 × 10^8^ CFU/mL and spread on the surface of the MRS agar and allowed to dry. Antibiotic disks were placed on the MRS agar surface and cultured for 24 h at 37°C, and the inhibition zone around each disk was measured. The following antibiotic discs (Hangzhou Microbial Reagent Co., LTD, China) were used: penicillin G (10 units), ampicillin (10 μg), tetracycline (30 μg), ceftriaxone (30 μg), chloramphenicol (30 μg), erythromycin (15 μg), clindamycin (2 μg), kanamycin (30 μg) and ciprofloxacin (30 μg).

### 2.2 *In vitro* studies on probiotic characteristics

#### 2.2.1 Growth and acid-producing curve curves

Detect the growth performance and acid production capacity of *P. pentosaceus* SNF15 (16). The cultured solutions of isolated strains were added to 80 mL MRS broth (1% v/v) and hermetically incubated at 37°C. The absorbances at 600 nm and pH were measured once every 2 h and continued until 24 h of culturing. Acid production and growth curves were plotted with time as the horizontal coordinate and pH or OD_600nm_ as the vertical coordinate.

#### 2.2.2 Antibacterial activity

Using the agar well diffusion method, test the antimicrobial activity of the strain isolate (17). To explore the antagonistic effect of the supernatant of 24 h cultures of *P. pentosaceus* SNF15 on *E. coli* K99 (Isolated from the feces of diarrheic calves), *P. pentosaceus* SNF15 was inoculated into MRS Broth at a 1% dose and cultured at 37°C for 24 h. Then, the supernatant was obtained by centrifuging the culture solution at 12000 r/min for 20 min.

Briefly, nutrient agar (Qingdao hopebiol, China) was poured on the culture dish as the first layer, and the Oxford cup was gently placed on the agar after solidification. Then, *E. coli* K99 cultured overnight was diluted to 1 × 10^6^ CFU/ mL using normal saline. Then, the diluted *E. coli* K99 was mixed with soft Mueller-Hinton Agar (Qingdao hopebiol, China) and poured on nutrient agar as the second layer. After the second layer solidification, remove the Oxford cup. 200 μL of the prepared supernatant of the isolated strain was added into each Oxford cup hole and cultured at 37°C for 12 h. MRS broth as a negative control and tested in triplicate.

#### 2.2.3 Bacteriostatic substance

Analyze the main bacteriostatic substances of *P. pentosaceus* SNF15 (18). Use the same way as in 2.2.2. to get *P. pentosaceus* SNF15 supernatant. Catalase or protease K or NaOH (Beijing Solarbio Science & Technology Co., Ltd., China) was added to the supernatant of *P. pentosaceus* SNF15. The final concentrations were adjusted to 1 mg/mL for protease K, 5 mg/mL for catalase, and pH 7.0, respectively. The catalase group was placed in 37°C water bath for 1 h; the protease K group was placed in 37°C water bath for 3 h. The same method was used as 2.2.2 to determine the inhibitory effect of the treated supernatant on *E. coli* K99. The pH of the supernatant of *P. pentosaceus* SNF15 was adjusted to 7.0 using NaOH (Beijing Solarbio Science & Technology Co., Ltd., China). The original supernatant was used as a positive control to assess the impact of treatment on the antibacterial active substances in the supernatant.

#### 2.2.4 Tolerance to bile salt and artificial gastroenteric fluid

Slightly modified according to Ye K’s method (19). The bile salt tolerance of *P. pentosaceus* SNF15 was assessed. To evaluate resistance to bile salt, a 5% inoculum size *P. pentosaceus* SNF15 solution, which had been cultured for 24 h, was inoculated into the MRS broth with 0.3% (w/v) bile salts, inoculated in common MRS broth as a control, and cultured at 37°C for 6 h.

Artificial gastroenteric fluid tolerance of *P. pentosaceus* SNF15 was assessed under gastrointestinal tract-like conditions. To prepare simulated gastric juice, MRS broth medium adjusted to pH 3.0 was added with 1 mg/mL pepsin (from Beijing Solarbio Science & Technology Co., Ltd., China) under sterile conditions. Similarly, to prepare artificial intestinal juice, 1 mg/mL of trypsin (1:250) (Beijing Solarbio Science & Technology Co., Ltd., China) was added to the sterile, pH 8.0 MRS broth. Inoculate into untreated MRS broth medium as a control. Fresh bacterial culture prepared in the same conditions mentioned above was cultured at 37°C for 4 h.

The absorbance at 600 nm was measured using a U-V spectrophotometer. The experiments were repeated thrice. The survival rate is calculated according to the following formula:


$$\mathrm{Survival rate}\;\left(\%\right)=\frac{N0}{N1}*100\%$$

N0 is the absorbance at 600 nm of *P. pentosaceus* SNF15 cultured in processed MRS broth, and N1 is the absorbance at 600 nm of *P. pentosaceus* SNF15 cultured in common MRS broth.

#### 2.2.5 Adhesion to Caco-2 cell

Adhesion of *P. pentosaceus* SNF15 to the Caco-2 cell line *in vitro* was performed as reported (20). Caco-2 cells were maintained in a basal medium composed of DMEM medium (Wuhan Pricella Biotechnology Co., Ltd., China) supplemented with 20% FBS (Meilunbio^®^, China) and 1%P/S (Beijing Solarbio Science & Technology Co., Ltd., China). Caco-2 cells were cultured at 37°C in an atmosphere containing 5% CO_2_. 300,000 Caco-2 cells were inoculated on the 12-well plate, and the experiment was started after the cell density grew to more than 80%. Caco-2 cells were washed twice using PBS and then infected with 1 × 10^8^ CFU/mL *P. pentosaceus* SNF15 in basal medium without P/S and FBS. The experimental setup also included one control: a negative control without *P. pentosaceus* SNF15. After 2 h, PBS washed away non-adherent bacteria, and Caco-2 cells were digested with pancreatic enzymes and collected in sterile tubes. Cell counts of the negative control were performed under a microscope. Colony counting was performed by using the viable plate counting method.

The experiments were repeated thrice. The *P. pentosaceus* SNF15 adheres to the Caco-2 cell line was calculated using the following formula:


$$\mathrm{Adhesion rate}\left(\%\right)=\frac{M1}{M0}*100\%$$

Where M1 is the number of adhesive strains (lgCFU/mL), and M0 is the cell count (lgCFU/mL) of the negative control.

### 2.3 Whole-genome sequencing of *Pediococcus pentosaceus* SNF15

According to the standard protocol provided by Oxford Nanopore Technologies (ONT), perform experimental procedures. Based on the manufacturer’s recommendations, use the TIANamp Bacteria DNA Kit to extract DNA from probiotics. Using a Nanodrop ND2000 spectrophotometer (ThermoFisher Scientific, Waltham, MA, USA) measure concentration and purity by using a DNA and a Qiagen DNA extraction kit (Qiagen, ThermoFisher, USA) with a Qubit fluorometer. Using 0.35% agarose gel electrophoresis, assess the integrity of the DNA sample. Then, the Qubit library will be measured and produced utilizing the ligation sequencing kit (SQK-LSK109 Ligation Sequencing Kit, Oxford Nanopore Technologies, Oxford, UK). After that, DNA damage and terminal repair were performed using magnetic bead purification to join and purify the DNA. Then, the PromethION sequencer was used to sequence the sample for the whole genome.

The filtered reads were assembled using Canu (version 1.5) software, the assembly results were corrected using the three-generation reads using Racon (version 3.4.3) software, and the cyclotron (version 1.5.5) software was used to calculate and adjust the starting site. Gene prediction was performed by the software Prodigal (version 2.6.3). To search for repeats in the genome, using the software Repeat Masker (version 4.0.5) compares the bacterial genome with a known repeat sequence database. The Rfam database includes the sequences of the above 5S rRNA, 16S rRNA, and 23S rRNA of many species. Based on these sequences, CMs carrying sequence and structure information for the three rRNAs were developed, respectively. Software Infernal (version 1.1.3) was used to predict the 3 types of rRNAs in the genome more accurately according to the covariance model. tRNAscan-SE v2.0 was used to predict tRNA in the genome.

### 2.4 Animal experiment

#### 2.4.1 Animal management and experimental procedures

In the current study, 30 half-male and half-female KM mice (5 weeks old, 30 ± 0.5 g initial body weight) purchased from SiPeiFu Biotechnology Co. LTD (Beijing, China) were raised in the Experimental Animal Center of Hebei Agricultural University under light-controlled (12-h light/dark cycle) and temperature-controlled (22 ± 1°C) conditions. The mice had free access to water and food.

*Pediococcus pentosaceus* SNF15 was statically cultured in MRS broth at 37°C for 24 h, and *E. coli* K99 was agitated cultured under 37°C 180 r/min for 12 h. Subsequently, *P. pentosaceus* SNF15 and *E. coli* K99 was harvested by centrifugation (4,000 r/min, 20 min) respectively. After washing twice using stroke-physiological saline solution, *P. pentosaceus* SNF15 was resuspended in an appropriate volume of stroke-physiological saline solution to a concentration of 1 × 10^9^ CFU/mL, and *E. coli* K99 was resuspended in an appropriate volume of stroke-physiological saline solution to a concentration of 1 × 10^8^ CFU/mL.

A total of 30 KM mice were randomly assigned to 5 groups, each containing 6 mice (*n* = 6): (I) CK group received 200 μL stroke-physiological saline solution for 14 days. (II) CN group received 200 μL *E. coli* K99 (1 × 10^8^ CFU/mL) for 1–7 days and stroke-physiological saline solution at 8–14 days. (III) CIP group received 200 μL *E. coli* K99 (1 × 10^8^ CFU/mL) at 1–7 days and 200 μL of 50 mg/kg Ciprofloxacin solution at 8–14 days. (IV) PPE group received 200 μL *P. pentosaceus* SNF15 solution (1 × 10^9^ CFU/mL) at 1–7 days and 200 μL *E. coli* K99 (1 × 10^8^ CFU/mL) at 8–14 days. (V) PTE group received 200 μL *E. coli* K99 (1 × 10^8^ CFU/mL) at 1–7 days and 200 μL *P. pentosaceus* SNF15 solution (1 × 10^9^ CFU/mL) at 8–14 days. All the administration methods were oral administration by gavage.

Based on Yang B et al. (21) develop fecal scoring criteria (Table 1) to evaluate the diameter of mice feces smudges.

**Table 1:** Fecal status score of mice.

Fecal scoring criteria				
Smudge diameter (cm)	<0.5	0.5 ~ 1	1 ~ 1.5	1.5 ~ 2
Score	1	2	3	4

The diarrhea rate of each group was calculated according to the formula. Body weight was recorded every 2 days, and fecal track scores were recorded every 3 days. On 15 days, after recording body weight, the mice were anesthetized and sacrificed by cervical dislocation. After blood was collected by eyeball enucleation, the weight of the liver, spleen, and small intestinal tissue was recorded, and then the jejunum tissues and cecal contents were collected. The diarrhea rate and weight change rate of mice were calculated according to the formula:


$$\mathrm{Diarrhea rate}\left(\%\right)=\frac{D1}{D0}*100\%$$

Where D1 and D0 are the diarrhea mice in this group and the total number of mice in this group.


$$\mathrm{Weight change rate}\left(\%\right)=\frac{W1}{W0}\times 100\%$$

Where W0 is the body weight of mice at 0 days and W1 is the body weight of mice at 2, 4, 6, 8, 10, 12, and 14 days, respectively.

#### 2.4.2 RNA extraction and quantitative real-time polymerase chain reaction

The total RNA of jejunum tissue was extracted by using TransZol Up Plus RNA Kit (TransGen Biotech, Beijing, China) and quantitatively analyzed using a NanoDrop D2000 instrument (Thermo Fisher Science, USA); the RNA was stored at −80°C. The mRNA was reverse-transcribed into cDNA using EasyScript^®^ One-Step gDNA Removal and cDNA Synthesis SuperMix (TransGen Biotech, Beijing, China) kit. The reverse transcription reaction conditions were genomic DNA elimination at 42°C for 15 min and inactivation of the reverse transcriptase at 85°C for 5 s. qPCR assays were performed to assess the relative expression levels of the IL-6, IL-1β, and TNF-α. The expression level of *GAPDH* was used as the reference gene. The amplification conditions were pre-denaturation at 95°C for 1 min, followed by 40 cycles of PCR reaction with denaturation at 95°C for 5 s and annealing at 60°C for 15 s. All primers were synthesized by Sangon Biotech Co., Ltd. (Shanghai, China). The specific primers for interleukin were IL-6 (5′-CTTCTTGGGACTGATGCTGGTGAC-3′; 5′-TCTGTTGGGAGTGGTATCCTCTGTG-3ʹ), IL-1β (5′-CCTGGGCTGTCCTGATGAGAG-3ʹ; 5ʹ-TCCACGGGAAAGACACAGGTA-3ʹ), TNF-α (5ʹ-GGACTAGCCAGGAGGGAGAACAG-3ʹ; 5ʹ-CAATGTGTCCGTCGTGGATCT-3ʹ), GAPDH (5ʹ-CAATGTGTCCGTCGTGGATCT-3ʹ; 5ʹ-GTCCTCAGTGTAGCCCAAGATG-3ʹ).

#### 2.4.3 Enzyme-linked immunosorbent assay

The jejunum samples were homogenized with pre-cooled PBS and were centrifuged at 12000 × *g* for 10 min at 4°C. Then, the collected supernatant was transferred to a sterile tube and used for ELISA analyses. Secretion levels of TNF-α, IL-6, and IL-1β in the jejunum samples homogenates were colorimetrically determined by enzyme-linked immunosorbent assay kits (mlbio, Shanghai, China) according to the manufacturer’s instructions.

#### 2.4.4 Hematoxylin–eosin staining in jejunum tissue

Use standard saline buffer to gently rinse the contents of jejunum tissue samples and place them into a 4% (v/v) paraformaldehyde fixative for fixation. Subsequently, embed jejunum tissue in paraffin and cut into 5 μm sagittal sections. The sections were deparaffinized with xylene and stained with hematoxylin and eosin (H&E). Images of jejunum tissue were captured using a light microscope (NIKON, Tokyo, Japan) to observe pathological structural changes.

#### 2.4.5 Immunohistochemistry detection of tight junction proteins and mucin

ZO-1, occludin, claudin-1, and MUC2 are crucial tight junction (TJ) proteins and mucin, contributing to intestinal physical barrier function (22). Immunofluorescence staining was performed on jejunum tissue. After antigen retrieval of jejunal sections, use 3% hydrogen peroxide to treat the sections, 0.3% Triton X-100 permeabilize and block with 3% BSA (Servicebio Technology, Wuhan, China) at room temperature for 30 min. Then, the sections were incubated overnight with primary antibodies directed against them. After incubation with the secondary antibody, stained sections in DAB (Servicebio Technology, Wuhan, China) and hematoxylin were observed under a microscope. Images were processed using ImageJ software.

#### 2.4.6 16S rRNA amplification and sequencing

Fresh content in the cecum was collected in a sterile tube. The contents were put in liquid nitrogen and then preserved at −80°C for the next step. The total DNA genome was extracted from colonic contents. PCR-amplified V3–V4 region of the 16S rRNA gene. Amplicons from all colon-content bacterial 16S rRNA samples were sent to Peisenor Biotechnology (Shanghai, China) for sequencing on an Illumina HiSeq 2500 platform. Data analysis was performed by using the Personalbio Cloud Platform.^1^ Analysis of alpha diversity (α-diversity), beta diversity (β-diversity), rarefaction curve, and taxonomic composition was performed by using QIIME2 software (version 2019.4). β-diversity was displayed by principal coordinates analysis (PCoA) with the help of R software, QIIME2 software, and ape bag.

### 2.5 Statistical analysis

The results are shown as average ± standard error (SEM). One-way analysis of variance (ANOVA) by IBM SPSS Statistics (version 27) was used to determine statistical significance. Figures were created using GraphPad Prism (version 8.0.2) and Adobe Illustrator (version 2024). Differences between samples were considered significant at *p* < 0.05.

## 3 Results

### 3.1 Probiotic isolate and its safe identification

**Figure 1:**
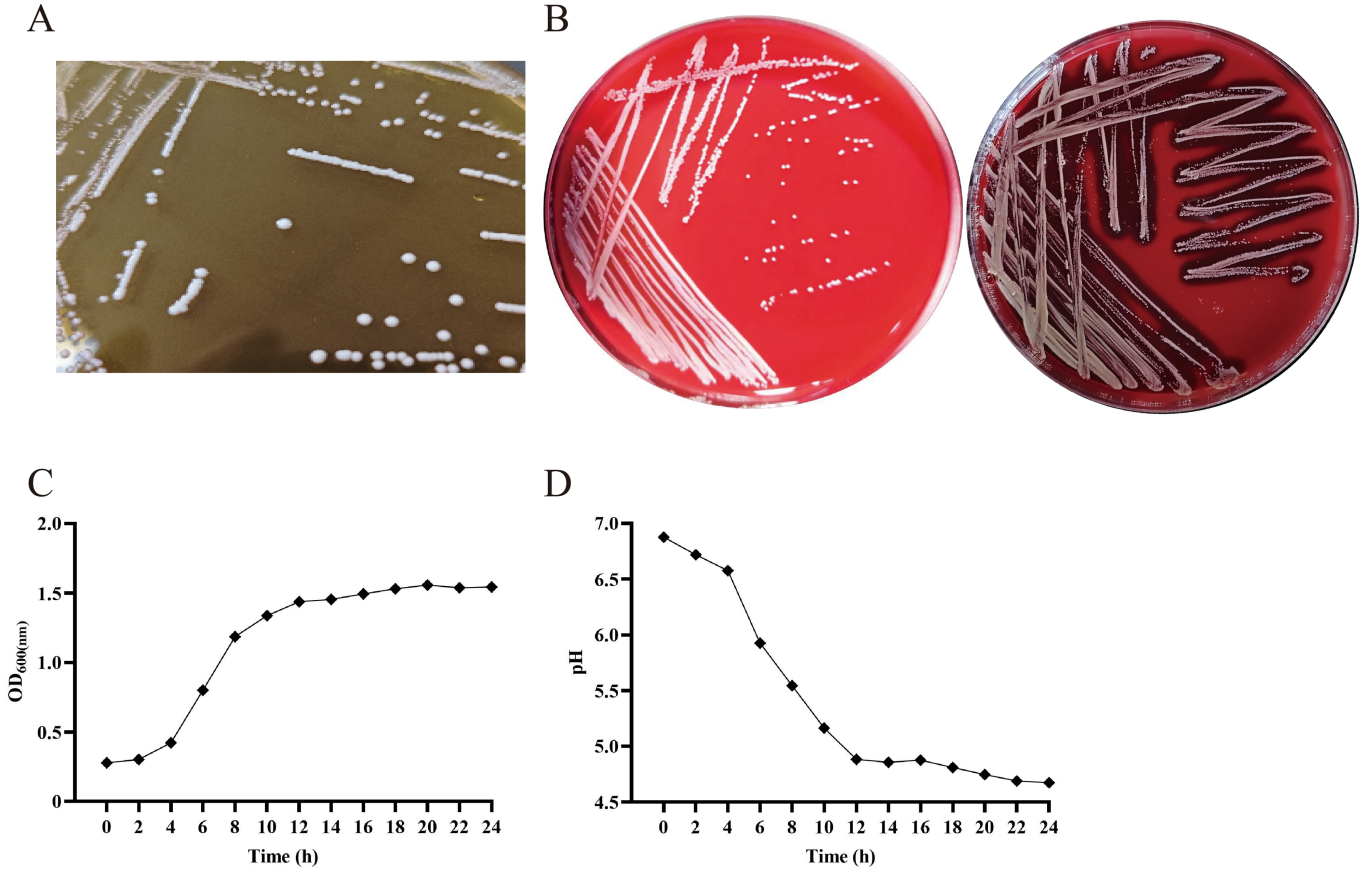
The colony form of *P. pentosaceus* SNF15 is not hemolytic and has good growth performance and acid production ability. **(A)** morphology of *P. pentosaceus* SNF15. **(B)** γ-hemolysis showed *P. pentosaceus* SNF15 (left), β-hemolysis showed by *S. aureus* (right). **(C)** Growth curve analysis of *P. pentosaceus* SNF15 was performed by measuring OD_600_. **(D)** The acid-producing curve of *P. pentosaceus* SNF15.

By comparing the results of the NCBI database with the BLAST tool, the isolated strain in our study was 100.00% similar to *Pediococcus pentosaceus*. Thus, the isolate was named *Pediococcus pentosaceus* SNF15 (*P. pentosaceus* SNF15). The colony morphology of *P. pentosaceus* SNF15 growth on MRS agar was white, circular, slightly elevated, and smooth with the entire margin (Figure 1A). The *P. pentosaceus* SNF15, following Gram staining, was identified as gram-positive Coccinea under the microscope.

#### 3.1.1 Hemolytic

The positive control of *S. aureus* (CVCC186158) showed an apparent β-hemolytic ring. While the *P. pentosaceus* SNF15 has no hemolytic ring and is judged as γ-hemolysis, indicating that the *P. pentosaceus* SNF15 cannot dissolve erythrocytes (Figure 1B).

#### 3.1.2 Antibiotics sensitivity

In the current study, *P. pentosaceus* SNF15 was assessed for antibiotic sensitivity properties. Antibiotic susceptibility tests showed that (Table 2) *P. pentosaceus* SNF15 was sensitive to penicillin G, ampicillin, tetracycline, ceftriaxone, chloramphenicol, doxycycline, erythromycin, and resistant to kanamycin and ciprofloxacin.

**Table 2:** Sensitivity of *P. pentosaceus* SNF15 to antibiotics.

Antibiotic	Sensibility
Penicillin G	S
Ampicillin	S
Tetracycline	S
Ceftriaxone	S
Chloramphenicol	S
Erythromycin	S
Clindamycin	S
Kanamycin	R
Ciprofloxacin	R

### 3.2 *In vitro* studies on *Pediococcus pentosaceus* SNF15 characteristics

#### 3.2.1 The growth character and acid-producing ability of the *Pediococcus pentosaceus* SNF15

The growth activity of *P. pentosaceus* SNF15 in MRS broth is shown in Figure 1C. *P. pentosaceus* SNF15 began entering the logarithmic growth phase after culturing for 2 h and approximately reached the stationary phase after 12 h. The acid-producing characteristic of *P. pentosaceus* SNF15 in MRS broth is shown in Figure 1D. The pH of *P. pentosaceus* SNF15 culture solution decreased with the passage of culture time and tended to be stable at about 4.67 after 12 h of culture.

#### 3.2.2 Bile salt and artificial gastroenteric fluid tolerance

Tolerance to the gastrointestinal environment is a prerequisite as a probiotic that mainly colonizes the gut. The survivability of *P. pentosaceus* SNF15 to 0.3% bile salt and artificial gastroenteric fluid was monitored. The results demonstrated that (Table 3) the survival rate of *P. pentosaceus* SNF15 in 0.3% bile salt is 26.02%, and the survival rate in artificial gastric juice and artificial intestinal juice is 50.56 and 55.62%, respectively.

**Table 3:** Gastrointestinal tolerance and *in vitro* adhesion of *P. pentosaceus* SNF15.

Probiotic properties	*P. pentosaceus* SNF15 (Average ± SEM)
0.3% bile salt tolerance (Survival rate, %)	26.022 ± 0.001
Simulated gastric fluid tolerance (Survival rate, %)	50.565 ± 0.003
Artificial intestinal juice tolerance (Survival rate, %)	55.620 ± 0.009
Adhere caco-2 ability (Adhesion rate, %)	13.930 ± 1.149

#### 3.2.3 Adhesion to Caco-2 cells of *Pediococcus pentosaceus* SNF15

We performed adhesion tests to evaluate the ability of *P. pentosaceus* SNF15 to colonize the gastrointestinal tract. As shown in Table 3, the adhesion rate of *P. pentosaceus* SNF15 on Caco-2 cells is 13.93%.

#### 3.2.4 Antibacterial activity and antibacterial substance of analysis

The inhibition effect of the *P. pentosaceus* SNF15 was tested using the agar well diffusion method against *E. coli* K99. The results are shown in Table 4. *P. pentosaceus* SNF15 showed the antibacterial effect of inhibition on the *E. coli* K99, and the inhibitory diameter is 19.10 ± 0.436 mm. The effect of different treatments on the antibacterial activity of *E. coli* K99 of *P. pentosaceus* SNF15 supernatant is shown in Table 4. The control is supernatant without treatment. NaOH was added to the *P. pentosaceus* SNF15 supernatant to exclude the effect of organic acid and adjust the pH to 7.0. The result shows that it did not affect the antibacterial activity (*p* > 0.05). To exclude the effect of bacteriocin on antibacterial activity, the supernatant was treated with protease K. The result showed that the antibacterial activity of the treatment group was significantly decreased from that of the control group (*p* < 0.05). The supernatant was treated with catalase to eliminate the effect of hydrogen peroxide. As a result, the antibacterial activity of the treatment group was significantly decreased from that of the control group (*p* < 0.05). These results indicate that the main bacteriostatic substances of *P. pentosaceus* SNF15 include bacteriocin and hydrogen peroxide.

**Table 4:** Antibacterial activity and antibacterial substance analysis of *P. pentosaceus* SNF15 against *E. coli* K99.

Pathogen	Treatment	Inhibition zone diameter (mm, Average ± SEM)
		*P. pentosaceus* SNF15
*E. coli* K99	Control	19.10 ± 0.44^a^
	pH	19.08 ± 0.40^a^
	Catalase	15.05 ± 0.14^b^
	Proteinase K	13.95 ± 0.44^b^

^a,b^ The database is expressed as the mean ± SEM. Significant differences were considered at *p* < 0.05. Different letters are marked next to data, without the same letter differ significantly (*p* < 0.05).

### 3.3 Complete genome analysis of *Pediococcus pentosaceus* SNF15

#### 3.3.1 Genome assembly result

The basic information on the genome of *P. pentosaceus* SNF15 is shown in Table 5. The genome of *P. pentosaceus* SNF15 consists of 1,895,681 bp with 37.33% GC content. The genome of *P. pentosaceus* SNF15 consists of 1 chromosome (contig0001) and 2 plasmids (contig0002 and contig0003), and the chromosome and plasmid genome are circular. The length of the chromosome is 1,842,076 bp. The length of 2 plasmids is 31,328 and 22,277 bp, respectively. It contains 1,855 protein-coding sequences, 1,668,747 bp, accounting for 88.02% of the total length; the average gene length is 899 bp, the max gene length is 8,601 bp, and the min gene length is 90 bp. The total repetitive sequence length is 4,435 bp, and the repetitive sequence content is 0.23%. The genome contains 15 rRNA genes and 56 tRNA genes. The predicted coding genes were functionally annotated by aligning them with 5 databases: Kyoto Encyclopedia of Genes and Genomes (KEGG), Gene Ontology (GO), evolutionary genealogy of genes Non-supervised Orthologous Groups (eggNOG), Carbohydrate-active enzymes (CAZy), and Comprehensive Antibiotic Research Database (CARD). Based on the protein sequence alignment, the annotated gene is compared with various databases to obtain the corresponding functional annotation information. Among them, 955 genes were annotated in the KEGG database, 1,466 genes were annotated in the GO database, 1,607 genes were annotated in the eggnog database, 57 genes related to carbohydrate activity were annotated by CAZy, and 1 drug resistance gene was annotated by CARD.

**Table 5:** Whole genome sequencing features of *P. pentosaceus* SNF15.

Features	Results	Features	Results
Scaffold length	1,895,681 bp	Max length	860,1 bp
GC content	37.33%	Min length	90 bp
Circular chromosome length	1,842,076 bp	Repeat sequence length	443,5 bp
Circular plasmid 1	313,28 bp	Repeat sequence content	0.23%
Circular plasmid 2	222,77 bp	5S rRNA	5
Total gene length	166,874,7 bp	16S rRNA	5
Proportion of coding genes	88.02%	23S rRNA	5
Number of genes	1855	tRNA	56
Average gene length	899 bp		

#### 3.3.2 KEGG annotation results

**Figure 2:**
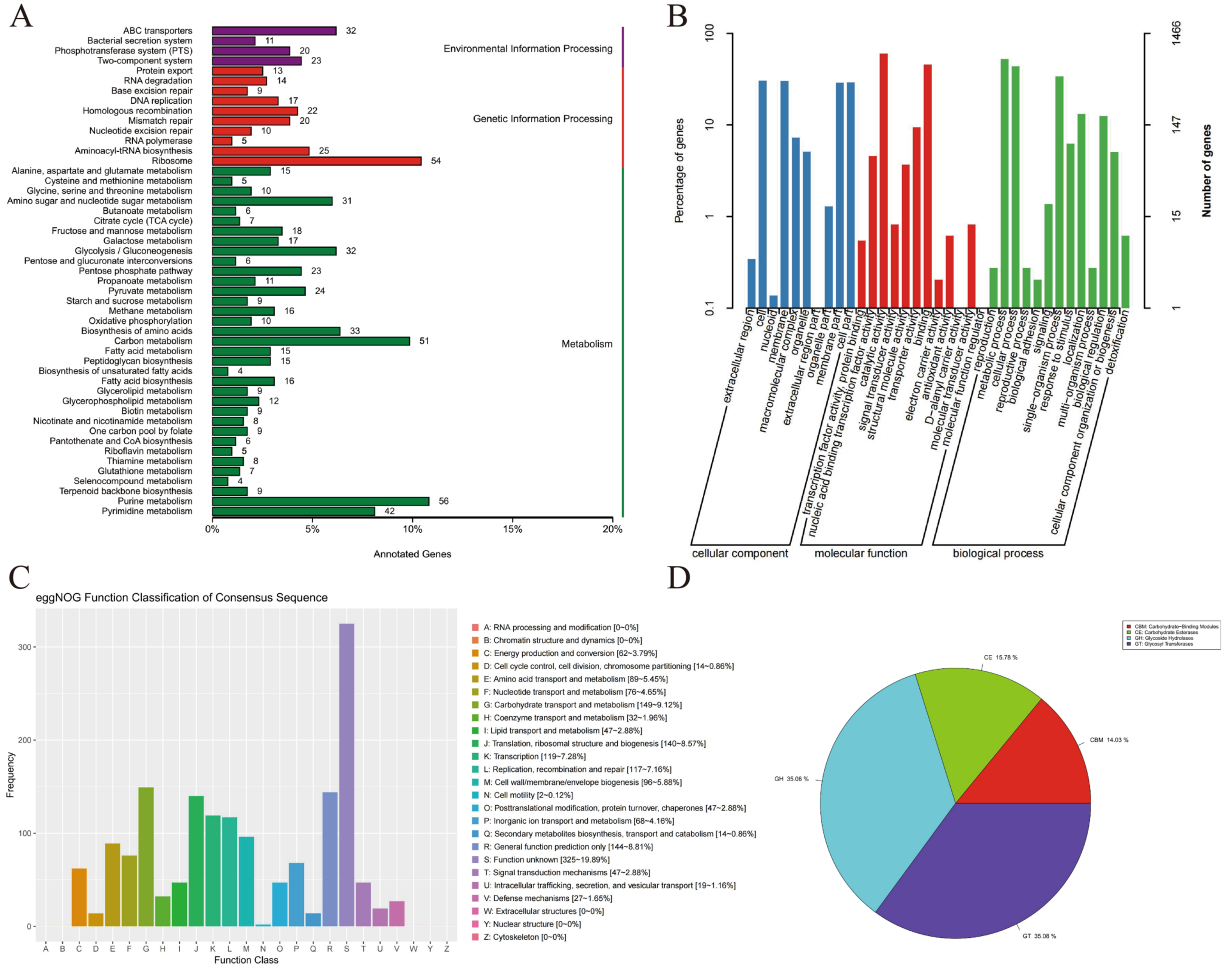
Complete genome of *P. pentosaceus* SNF15. **(A)**: KEGG databases annotation result classification chart. **(B)**: GO databases annotation result classification chart. **(C)**: eggNOG databases annotation result classification chart. **(D)**: CAZy databases annotation result classification chart.

955 genes were annotated in the KEGG database, accounting for 51.48% of the total of *P. pentosaceus* SNF15 (Figure 2A). These genes were annotated with four categories (environmental information processing, genetic information processing, cellular Processes, and metabolism), including nineteen level 2 KEGG pathways and 76 “ko-” related reference pathways of metabolism annotated. Among the four categories of KEGG pathways, metabolism contains the most significant number of genes (558 genes), including level 2 pathways of 12 metabolism and 64”ko-” related pathways. In the level 2 pathways about metabolism, nucleotide metabolism (gene number: 80), carbohydrate metabolism (gene number: 121), and global and overview maps (gene number: 275) are the top three pathways. Carbohydrate metabolism mainly contains a total of 14 pathway information, including glycolysis/gluconeogenesis (ko00010), amino sugar and nucleotide sugar metabolism (ko00520), pyruvate metabolism (ko00620), pentose phosphate pathway (ko00030), and the number of related genes is 32, 31, 23, and 24, respectively. Nucleotide metabolism contains 2 pathways: purine metabolism (ko00230) has 56 genes annotated, and Pyrimidine metabolism (ko00240) has 42 genes annotated. Global and overview maps mainly consist of metabolic pathways, biosynthesis of secondary metabolites, and biosynthesis of antibiotics. It is worth noting that some pathways function as probiotic markers, such as terpenoid backbone biosynthesis, taurine and hypotaurine metabolism, and streptomycin biosynthesis (Table 6). These probiotic genes are an essential prerequisite for *P. pentosaceus* SNF15 to exert probiotic functions.

**Table 6:** *Pediococcus pentosaceus* SNF15 genome antibacterial, anti-inflammatory pathway and related genes.

No	Passway ID	Description	Gene number
1	ko00521	Streptomycin biosynthesis	3
2	ko00622	Xylene degradation	2
3	ko00362	Benzoate degradation	2
4	ko00900	Terpenoid backbone biosynthesis	9
5	ko00760	Nicotinate and nicotinamide metabolism	8
6	ko00430	Taurine and hypotaurine metabolism	4
7	ko00130	Ubiquinone and other terpenoid-quinone biosynthesis	1
8	ko01054	Nonribosomal peptide structures	1
9	ko00590	Arachidonic acid metabolism	1
10	ko00121	Secondary bile acid biosynthesis	1
11	ko01130	Biosynthesis of antibiotics	82

#### 3.3.3 GO annotation results

Annotation of coding genes against the GO database (Figure 2B). Gene functions could be classified into all three categories: biological process (BP), cellular component (CC), and molecular function (MF). 1,466 gene entries were annotated in the GO classification, accounting for 79.03% of all coding genes (1855) for 4,770 functions. The analysis revealed that 333 genes were enriched in CC terms, accounting for 22.71% of all GO coding genes. 445 and 443 genes were annotated, respectively, mainly focusing on cell and membrane. Others include extracellular region, nucleoid, macromolecular complex, organelle, etc. The analysis revealed that 938 genes were enriched in MF, accounting for 63.98% of all GO coding genes. 882 and 669 genes were annotated, respectively, mainly focusing on catalytic activity and binding. It also annotates nucleic acid binding transcription factor activity, signal transducer activity, transporter activity, etc. It is worth noting that 9 genes for antioxidant activity were annotated. The analysis revealed that 195 genes were enriched in BP terms, accounting for 13.30% of all GO coding genes. 771 genes were related to the metabolic process, and 641 were associated with the cellular process. The analysis also annotates single-organism processes, localization, biological regulation, response to stimulus, etc. It is worth noting that there are some functions, including reproduction, biological adhesion, and biological regulation, with 4 and 3 genes annotated, respectively.

#### 3.3.4 eggNOG annotation results

Annotation of coding genes against the eggNOG database (Figure 2C) subsequently identified 1,607 genes, which accounted for 86.63% of the total of *P. pentosaceus* SNF15. The eggNOG annotation results indicated that the primary known genes of *P. pentosaceus* SNF15 are in carbohydrate transport and metabolism (149 genes, accounting for 9.12%), translation, ribosomal structure and biogenesis (140 genes, accounting for 8.57%), transcription (119 genes, accounting for 7.28%), and replication, recombination and repair (117 genes, accounting for 7.16%).

#### 3.3.5 CAZy annotation result

In the CAZy database, the 57 genes were annotated into 4 functional classifications (Figure 2D). Among those genes, 20 genes were annotated as Glycoside Hydrolases (GH), 20 genes were annotated as glycosyl transferases (GT), 9 genes were annotated as carbohydrate esterases (CE), and 8 genes were annotated as carbohydrate-binding modules (CBM), which account for 35.08, 35.08, 15.78, and 14.03%, respectively.

#### 3.3.6 CARD annotation result

Compared with the CARD database (Table 7), as indicated by the results, the genome alignment of *P. pentosaceus* SNF15 identified 1 gene related to antibiotic resistance named OXA-224 (gene ID: GE000281), which causes penam; cephalosporin inactivation.

**Table 7:** Prediction of virulence factor of *P. pentosaceus* SNF15.

ARO_name	ARO_description	Resistance	Resistance mechanism
OXA-224	OXA-224 is a beta-lactamase found in *Pseudomonas aeruginosa*	Penam; cephalosporin	Antibiotic inactivation

### 3.4 Animal tests

#### 3.4.1 *Pediococcus pentosaceus* SNF15 attenuated damages in the physiological indexes of *Escherichia coli* K99-infection mice

**Figure 3:**
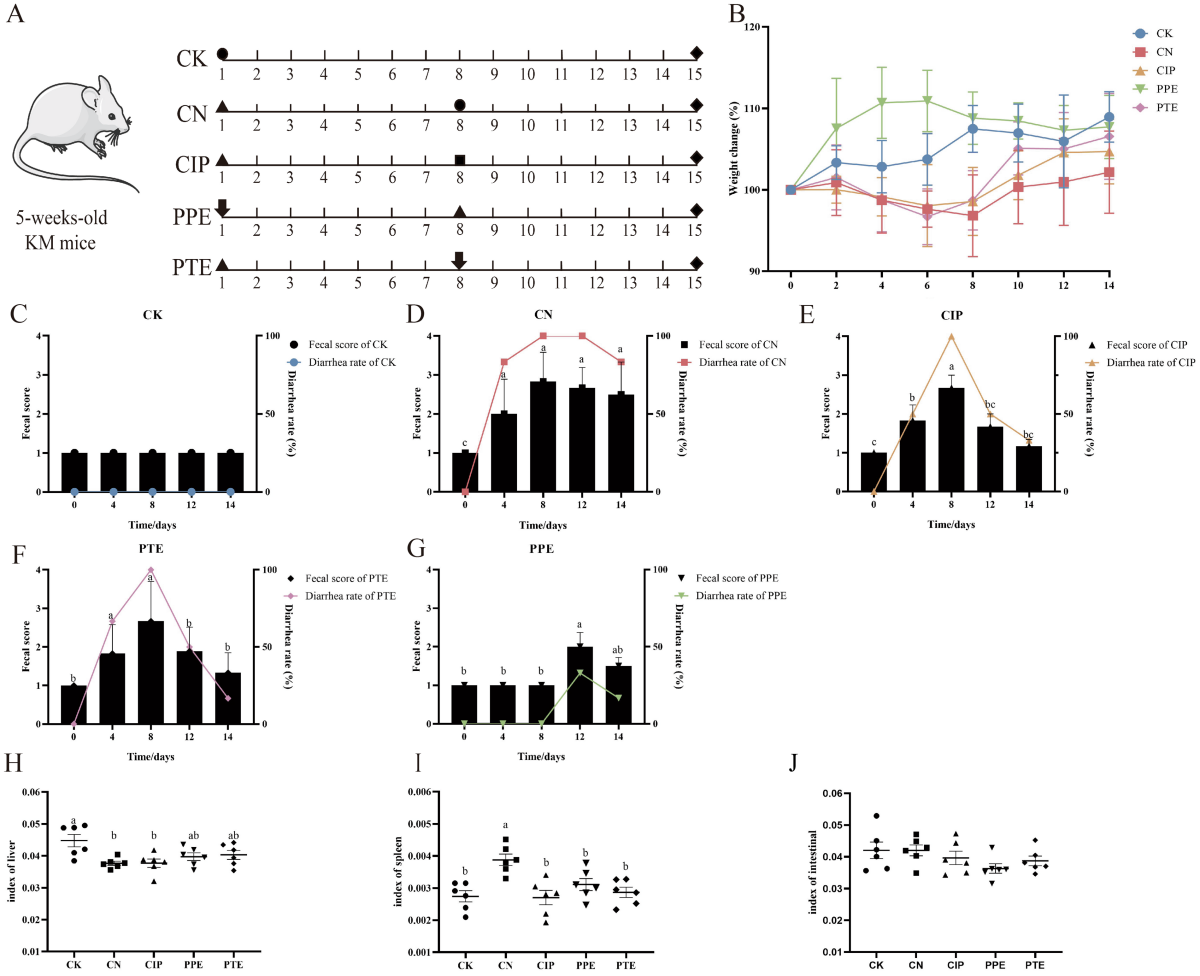
*P. pentosaceus SNF15* reduced clinical signs in *E. coli* K99 (*n* = 6). **(A)**: Diagram of the mice mode. The negative control group (CK), positive group (CN), Ciprofloxacin treatment group (CIP), *P. pentosaceus* SNF15 prevention group (PPE), and *P. pentosaceus* SNF15 treatment group (PTE) are indicated. Circles represent the gavaged normal saline; triangles represent the gavaged *E. coli* K99; squares represent the gavaged Ciprofloxacin; arrows represent the gavaged *P. pentosaceus* SNF15; rhombus represent the harvest time. **(B)**: Weight change for each group (*n* = 6). **(C)**: The fecal score and diarrhea rate for the CK group during the trial. **(D)**: The fecal score and diarrhea rate for the CN group during the trial **(E)**: The fecal score and diarrhea rate for the CIP group during the trial. **(F)**: The fecal score and diarrhea rate for the PPE group during the trial. **(G)**: The fecal score and diarrhea rate for the PTE group during the trial. **(H)**: The index of liver for each group (*n* = 6). **(I)**: The index of spleen for each group (*n* = 6). **(J)**: The index of intestinal for each group (*n* = 6). The database is expressed as the mean ± SEM. Significant differences were considered at *p* < 0.05. Different letters are marked at bars, without the same superscripts differ significantly (*p* < 0.05).

The experimental design in mice is shown in Figure 3A. Figure 3B shows the entire weight change of mice during the experiment. The body weight change of the CK group increased slowly during the trial for 1–14 days. The body weight change of the CN, CIP, and PTE groups was reduced rapidly during gavaged *E. coli* K99 for 1–7 days. The body weight change of the PPE group showed an increased trend during gavaged *P. pentosaceus* SNF15 for 1–7 days. At 8–14 days, the body weight change of the CN, CIP, and PTE groups slowly increased due to treatment with ciprofloxacin or *P. pentosaceus* SNF15, and the body weight growth trend for the CIP and PTE groups was better than the CN group. The body weight change of the PPE group showed a slowly decreasing trend but was still superior to the CN group.

Figures 3C–G show the fecal status score and diarrhea rate of all groups. The CK group had no diarrhea during the trial. The fecal status score and diarrhea rate of the CN, CIP, and PTE groups showed an increased trend. After treatment with ciprofloxacin or *P. pentosaceus* SNF15, both fecal status score and diarrhea rate significantly decreased. During only gavage for 1–7 days, the PPE group had no diarrhea, and some diarrhea appeared after gavage *E. coli* K99.

The organ index of mice was analyzed. Compared with the CK group, the liver shows considerable atrophy in the CN group (*p* < 0.05). The CIP, PPE, and PTE groups have no significant difference from the CN group (*p* > 0.05), and the liver index of PTE and PPE groups appears to be a rising trend and has no significant difference from the CK group (*p* > 0.05) (Figure 3H). Compared with the CK group, splenomegaly was found in the CN group (*p* < 0.05), and the CIP group of ciprofloxacin treatment, *P. pentosaceus* SNF15 prevention, and *P. pentosaceus* SNF15 treatment has no significant difference (*p* > 0.05) with the CK group (Figure 3I). There was no significant difference in intestinal coefficient (Figure 3J).

#### 3.4.2 *Pediococcus pentosaceus* SNF15 attenuated damages in the mechanical intestinal barrier of *Escherichia coli* K99-infection mice

**Figure 4:**
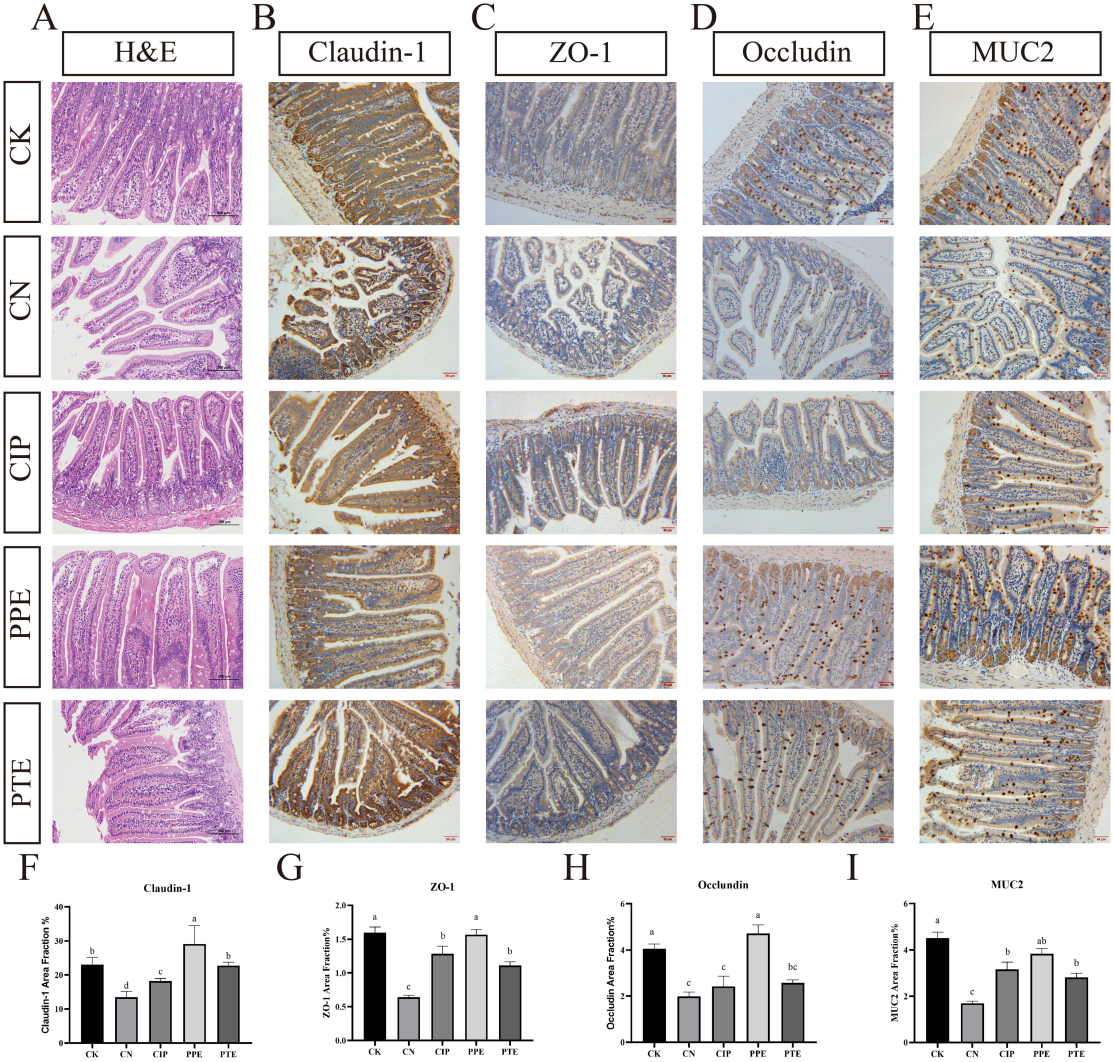
Gavaged *P. pentosaceus* SNF15 and Ciprofloxacin reduced the damage caused by *E. coli* K99 stimulation. **(A)** H&E staining of the jejune tissues. **(B)** Representative immunohistochemical staining of Claudin-1 in jejune tissue for each group. **(C)** Representative immunohistochemical staining of ZO-1 in jejune tissue for each group. **(D)** Representative immunohistochemical staining of occludin in jejune tissue for each group. **(E)** Representative immunohistochemical staining of MUC2 in jejune tissue for each group. **(F)** The claudin-1 area fraction for each group **(G)** The ZO-1 area fraction for each group. **(H)** The occludin area fraction for each group **(I)** The MUC2 area fraction for each group. Brown is immunopositive, and blue is immunonegative. The database is expressed as the mean ± SEM. Significant differences were considered at *p* < 0.05. Different letters are marked at bars, without the same superscripts differ significantly (*p* < 0.05).

The results of HE staining of jejunal tissue are shown in Figure 4A. The intestinal villi of the CK group were clear and intact in structure, and the epithelial cells were arranged neatly. There was no edema within the lamina propria. Jejunal intestinal villi in the CN group were severely damaged, and there was prominent edema within the lamina propria of the intestinal villi. Compared with the CN group, the intervention of *P. pentosaceus* SNF15 and ciprofloxacin can significantly alleviate the intestinal damage caused by *E. coli* K99.

Figure 4 showed that, due to the stimulation of *E. coli* K99, compared with the CK group, claudin-1, ZO-1, occludin, and MUC2 in the CN group showed a notable decrease (*p* < 0.05). However, oral administration of *P. pentosaceus* SNF15 in advance significantly improved the expression of claudin-1, MUC2, occludin, and ZO-1 (*p* < 0.05), and the expression of claudin-1 was significantly increased compared with the CK group (*p* < 0.05). After infection with *E. coli* K99, the expression of *P. pentosaceus* SNF15, Claudin-1, MUC2, and ZO-1 in oral administration increased significantly compared with the CN group (*p* < 0.05), and the expression level of occludin did not change significantly (*p* > 0.05). Oral ciprofloxacin increased the expression of claudin-1, MUC2, and ZO-1 (*p* < 0.05), while the expression of occludin had no significant change (*p* > 0.05).

#### 3.4.3 *Pediococcus pentosaceus* SNF15 attenuated damages in the inflammatory properties’ intestinal barrier of *Escherichia coli* K99-infection mice

**Figure 5:**
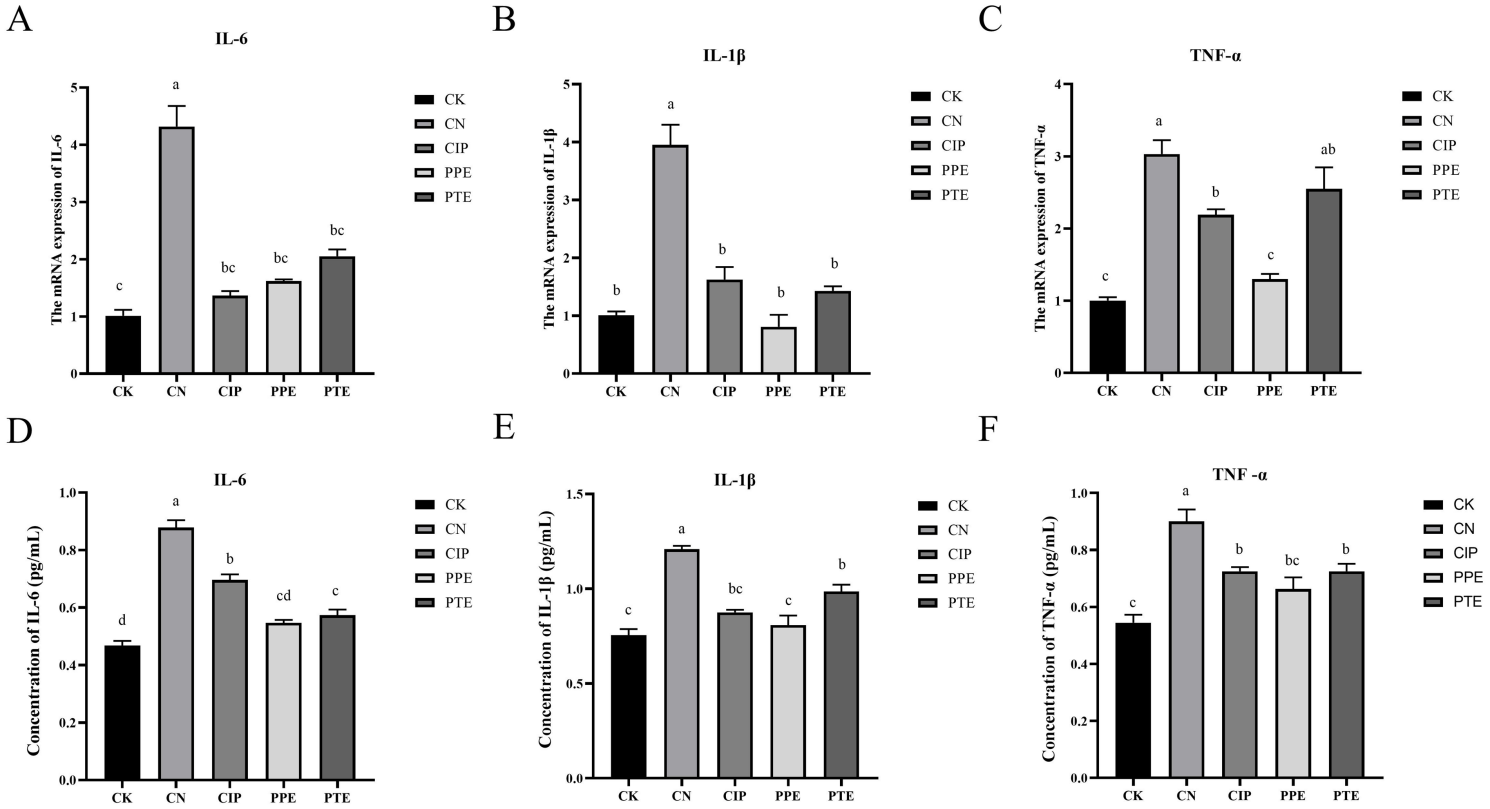
Gavaged *P. pentosaceus* SNF15 and Ciprofloxacin reduced inflammatory factor level of jejunum. **(A)**: The mRNA expression level of IL-6 in jejunum for each group. **(B)**: The mRNA expression level of IL-1β in jejunum for each group. **(C)**: The mRNA expression level of TNF-α in jejunum for each group. **(D)**: The secretion level of IL-6. **(E)**: The secretion level of IL-1β. **(F)** The secretion level of TNF-α.

We detect expression mRNA levels of IL-6, IL-1β, and TNF-α in the jejunum to assess the anti-inflammatory properties. The expression mRNA levels of IL-6, IL-1β, and TNF-α in the intestines of mice infected with *E. coli* K99 were significantly up-regulated (Figures 5A–C). The mRNA expression levels of IL-6, IL-1β, and TNF-α in the CIP group were significantly reduced compared with the CN group (*p* < 0.05), and the mRNA expression levels of IL-6, and IL-1β had no significant difference with the CK group (*p* > 0.05). Compared with the CK group, the expression mRNA levels of TNF-α elevated significantly (*p* < 0.05). The mRNA expression levels of IL-6, IL-1β, and TNF-α in the PPE group were significantly decreased compared with the CN group (*p* < 0.05) and had no significant difference compared with the CK group (*p* > 0.05). The mRNA expression levels of IL-6 and IL-1β in the PTE group were significantly reduced compared with the CN group (*p* < 0.05), and TNF-α showed a downward trend but no significant difference (*p* > 0.05).

The secretion levels of IL-6, IL-1β, and TNF-α in jejunum tissues of each group were detected by ELISA (Figures 5D–F). The CN group has the same trend with expression mRNA levels, which is significantly decreased compared with the CK group (*p* < 0.05). The secretion levels of IL-6, IL-1β, and TNF-α in the CIP, PPE, and PTE groups were significantly reduced compared with the CN group (*p* < 0.05). The secretion levels of IL-6 and TNF-α in PPE group, secretion levels of IL-1β in CIP group had no significant difference with the CK group (*p* > 0.05).

#### 3.4.4 *Pediococcus pentosaceus* SNF15 attenuated damages in the intestinal microbial barrier of *Escherichia coli* K99-infection mice

**Figure 6:**
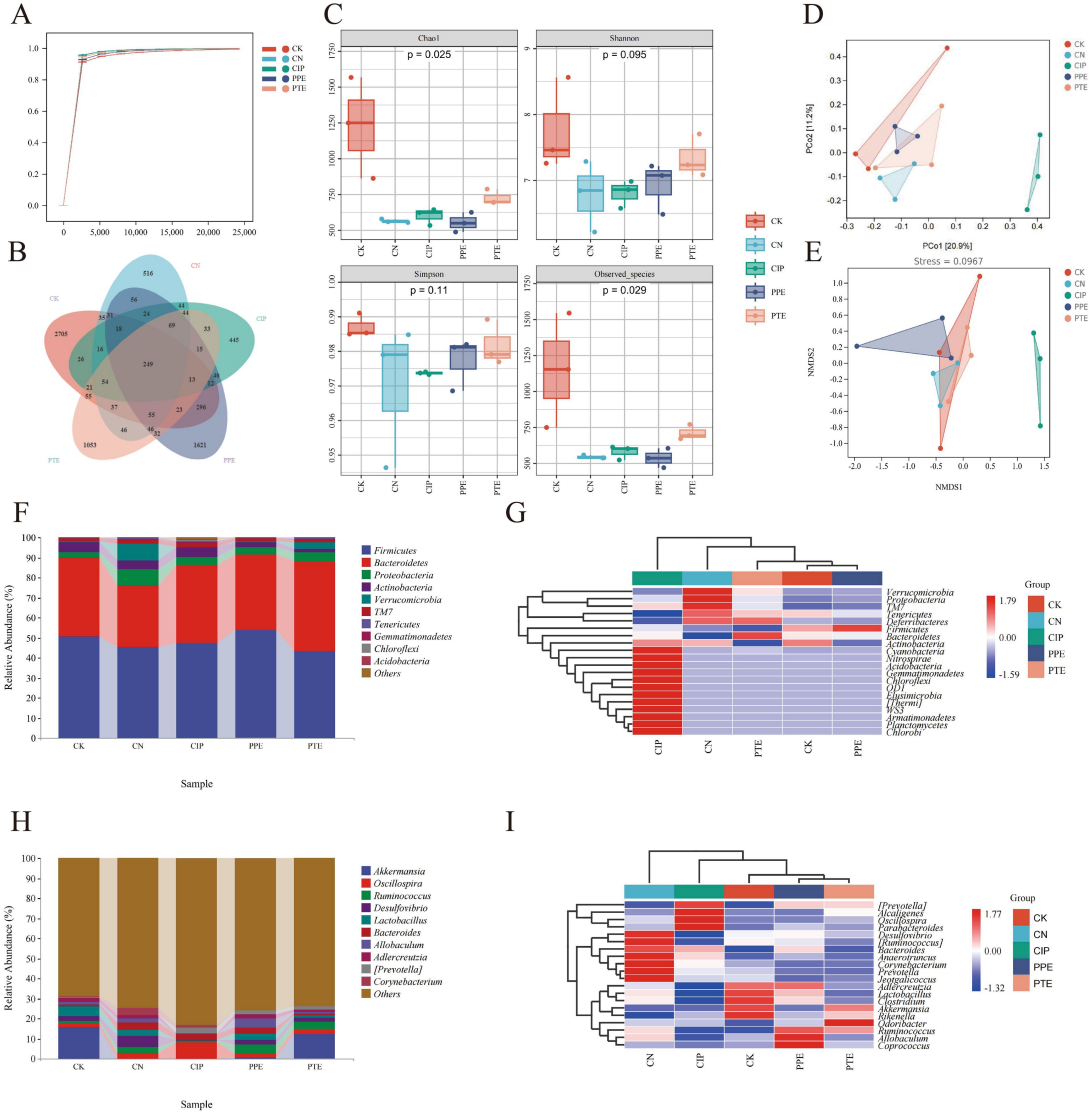
Gavaged *P. pentosaceus* SNF15 reduced the dysregulation of intestinal flora caused by *E. coli* K99 stimulation. **(A)** Goods coverage rarefaction curve. **(B)** Venn diagram analysis. **(C)** α-diversity for each group. **(D)** PCoA plot illustrating the overall expression pattern differences between each group. **(E)** Based on Bray_Curtis distance, NMDS maps of the microbial community in the intestinal flora. **(F)** Intestinal flora compositions at phylum level between groups. **(G)** A heat map of differential phylum level distribution is displayed for each group. The color gradient ranges from blue (low expression) to red (high expression), indicating changes in phylum level distribution. **(H)** Intestinal flora compositions at genus level between groups. **(I)** A heat map of differential genus level distribution is displayed for each group. The color gradient ranges from blue (low expression) to red (high expression), indicating changes in phylum level distribution.

In this research, 5 groups, including 15 samples, were subjected to high-throughput sequencing analysis. We obtain original data 1,001,103. After filigreeing and denouncing the original data, 879,706 effective-quality sequences were obtained from the 15 samples, and further, through merging and removal of chimeras, 524,424 high-quality sequences were obtained. The curves of good coverage index rarefaction in KM mice were flat and displayed saturated curves, demonstrating that the sequencing depth was sufficient in the current experiment (Figure 6A).

##### 3.4.4.1 *Pediococcus pentosaceus* SNF15 regulates the diversity of caecum microbiota in *Escherichia coli* K99-induced diarrhea mice

In total, we found 6,006 OTUs in all 15 samples (Figure 6B), of which 2,705 OTUs were unique in the CK group, 516 in the CN group, 445 in the CIP group, 1,621 in the PPE group, and 1,053 in the PTE group. The total number of OTUs common among all five groups was 249. Analysis of α-diversity (Figure 6C) showed a significant difference in the chao1 and observed species index between 5 groups, and the CK group has the highest diversity. The Simpson and Shannon indexes were not significantly different between the 5 groups (*p* > 0.05). However, the Simpson and Shannon indexes have increased between CK, PPE, PTE, and CK, CIP. PCoA (Figure 6D) and NMDS (Figure 6E) analyses were performed to assess the β-diversity. The samples from the CK and CIP groups exhibit relatively large distances, which shows that the overall community composition has changed between them.

##### 3.4.4.2 *Pediococcus pentosaceus* SNF15 regulates the composition of caecum microbiota in *Escherichia coli* K99-induced diarrhea mice

Accumulating evidence has suggested that dysregulation of gut microbiota is a critical factor in diarrhea. At the phylum level, microbiota displayed different abundance tendencies concerning different treatments. In total, we identified 10 bacterial phylums. The 5 dominant phylums for all samples were the Firmicutes, Bacteroidetes, Proteobacteria, and Actinobacteria. This phylum constituted the core of the microbiota and accounted for more than 90% of the taxonomic groups identified. The predominant phyla in different groups were as follows (Figure 6F): *Firmicutes* (50.67%), *Bacteroidetes* (38.99%), *Proteobacteria* (3.16%), *Actinobacteria* (4.88%) in the CK group; *Firmicutes* (445.19%), *Bacteroidetes* (30.90%), *Proteobacteria* (7.96%), *Actinobacteria* (4.35%) in the CN group; *Firmicutes* (47.41%), *Bacteroidetes* (38.65%), *Proteobacteria* (4.20%), *Actinobacteria* (4.75%) in the CIP group; *Firmicutes* (53.85%), *Bacteroidetes* (37.67%), *Proteobacteria* (3.46%), *Actinobacteria* (2.51%) in the PPE group; *Firmicutes* (43.46%), *Bacteroidetes* (44.80%), *Proteobacteria* (4.28%), and *Actinobacteria* (1.86%) in the PTE group. The heat map (Figure 6G) showed that *Firmicutes* in the CK and PPE groups were higher than in the CN, CIP, and PTE. The mean abundance levels of *Bacteroidetes* in the CN group are worse than those in the CK group, and levels of Bacteroidetes in the CIP, PPE, and PTE groups are higher than in the CN group. In contrast, the abundance of *Proteobacteria* in the CN group was higher than in the other 4 groups.

At the genus level, the changes in 10 genus were shown (Figure 6H): *Akkermansia* (15.53%), *Oscillospira* (2.04%), *Ruminococcus* (1.11%), *Desulfovibrio* (2.44%), *Lactobacillus* (5.14%) in the CK group; *Akkermansia* (0.01%), *Oscillospira* (3.07%), *Ruminococcus* (2.89%), *Desulfovibrio* (5.40%), *Lactobacillus* (2.86%) in the CN group; *Akkermansia* (0.01%), *Oscillospira* (7.99%), *Ruminococcus* (1.01%), *Desulfovibrio* (0.23%), *Lactobacillus* (0.13%) in the CIP group; *Akkermansia* (0.67%), *Oscillospira* (2.05%), *Ruminococcus* (4.21%), *Desulfovibrio* (2.64%), *Lactobacillus* (3.34%) in the PPE group; *Akkermansia* (12.23%), *Oscillospira* (2.71%), *Ruminococcus* (3.52%), *Desulfovibrio* (2.00%), *Lactobacillus* (1.13%) in the PTE group. The heat map (Figure 6I) showed a higher abundance of *Akkermansia* in the CK and PTE groups, a lower abundance in the CK and CIP groups, an abundance of *Oscillospira* in the CN and CIP groups higher than the other 3 groups, a higher abundance of *Ruminococcus* in the PPE and PTE groups, a higher abundance of *Desulfovibrio* in the CN group, and an abundance of *Lactobacillus* in the CK and PPE groups higher than the other 3 groups.

## 4 Discussion

Intestinal microflora has the potential to have an immunoregulatory function. Probiotics are nonpathogenic and beneficial for health microorganisms extracted and isolated from gut microbiota, which could improve the gut microbial population, reduce inflammatory markers, and perform other immunologic functions (23). Some research indicates that lactic acid bacteria (LAB) have been used to prevent and cure gastrointestinal illnesses and inflammation owing to their excellent biological efficacy and safety. LAB is also a proposed novel anti-inflammatory medicine (24). Therefore, *P. pentosaceus* SNF15, which is isolated from the feces of the healthy calf, its biological characteristics, safety, and whole genome sequencing were assessed in this study. Additionally, results suggest that *P. pentosaceus* SNF15 can reduce the clinical symptoms of mice diarrhea induced by *E. coli* K99, the expression level of inflammatory factors, and the state of intestinal microflora, and affect other functions. Probiotics have potential biological characteristics and safety, such as anti-pathogen activity, bile salt, gastrointestinal fluid tolerance, no hemolysis, antibiotic sensitivity, or other traits (25). The supernatant of *P. pentosaceus* SNF15 exhibited good antibacterial activity against *E. coli* K99 *in vitro*, which may be attributed to the production of hydrogen peroxide and organic acids. The two primary factors affecting the survival of LAB in the host’s gastrointestinal tract are bile salt secreted from the duodenum and enterogastric juice in the stomach and intestine (26). The ability of probiotics to effectively function in the gastrointestinal tract is a critical determinant for their selection as probiotics (27). In our study, *P. pentosaceus* SNF15 exhibited better tolerance in the gastrointestinal tract: the survival rate in bile salt, simulated gastric juice, and intestinal juice are 26.02, 50.56, and 55.62%, respectively. Besides, *P. pentosaceus* SNF15 will thrive in a suitable environment. Subsequently, it can lower the pH of the surrounding environment, inhibiting the colonization of some pathogens. Since probiotics are intended to colonize and attach to the host gut, which can prevent pathogen colonization, one of the assessed aspects is their ability to adhere to intestinal cells (28). *P. pentosaceus* SNF15 exhibited a 13.93% adherence rate to Caco-2 cells, similar to Pinto et al. (29) found a similar result for *P. pentosaceus* CFF4, which shows 11% adhesion to Caco-2 cells. Evaluating the antimicrobial resistance profile is a crucial factor in assessing potential probiotics, as these microorganisms may transmit antibiotic-resistance genes to harmful bacteria through direct or indirect means (30). *P. pentosaceus* SNF15 is sensitive to penicillin G, ampicillin, tetracycline, ceftriaxone, chloramphenicol, erythromycin, and clindamycin, resistant to kanamycin and ciprofloxacin. Hemolysis is used as an exclusion criterion in the screening of probiotics (31), and *P. pentosaceus* SNF15 is judged as γ-hemolysis that has no hemolytic ring. These results are consistent with Dowarah et al. (32). These results suggest that *P. pentosaceus* SNF15 may be combined with vancomycin or ciprofloxacin.

To access the probiotic potential and traits of *P. pentosaceus* SNF15 more comprehensively, analyzing whole genome sequencing of *P. pentosaceus* SNF15 is obligatory. The genome sequence analysis of *P. pentosaceus* SNF15 indicates that the genome length of *P. pentosaceus* SNF15 was 1,895,681 bp. The GC content was 37.33%. The bacterium contained 2 circular plasmids of 31,328 bp and 22,277 bp, respectively. The genome size and GC content of *P. pentosaceus* SNF15 showed similarity with the previously reported *P. pentosaceus* strains isolated from various sources (33). The functional annotation by the eggNOG database for *P. pentosaceus* SNF15 indicated 1,607 genes classified into 20 functional categories, with the function-unknown genes accounting for 19.89%. The large number of unknown function genes discloses the uniqueness and unknown potential of *P. pentosaceus* SNF15. In this research, pathway enrichment analysis indicated that vast genes were enriched in carbohydrate metabolism, which means *P. pentosaceus* SNF15 has a strong capacity for carbohydrate metabolism. This trait is desirable for a probiotic strain. Similar findings were reported by Liu et al. for their strain (34). This forebode *P. pentosaceus* SNF15 may be able to inhabit a broad range of environmental niches. We discovered that the pathway for biosynthesis of streptomycin and other antimicrobial substances was annotated in the *P. pentosaceus* SNF15 whole genome sequence, contributing to competitive colonization with the pathogen. Furthermore, we also found a large number of genes related to immune, antioxidant, anti-inflammatory, and antibacterial genes encoded by *P. pentosaceus* SNF15, including arachidonic acid, ubiquinone and other terpenoid-quinone, secondary bile acid, nicotinate and nicotinamide, terpenoid backbone, taurine and hypotaurine and other pathways related to the inflammatory response. Some studies indicated that arachidonic acid metabolism is associated with COX1, COX2, and LTA4 targets. Arachidonic acid metabolites are essential to safeguard the function of the immune system and resolve inflammation, and they play a crucial role in inflammatory disorders (35). Nicotinate and nicotinamide metabolism have been annotated similarly. Nicotinamide (NAM) and nicotinic acid (NA) are the main representatives of vitamin B3. NAM is nicotinamide adenine dinucleotide (NAD+) (36). Secondary bile acids in fecal levels of diarrhea patients are reduced; secondary bile acids regulate intestinal flora and intestinal permeability, which is essential to holistic health (37, 38). *P. pentosaceus* SNF15 is involved in the biosynthesis of secondary bile acids and the metabolism of nicotinic acid nicotinamide, and we hypothesize that it contributes to the rebuilding of intestinal flora and maintenance of the intestinal mechanical barrier. Xylene, as a volatile organic compound, can escape into the environment from oil and gas operations and manufacturing industries and may lead to chronic obstructive pulmonary disease, posing significant health risks to animals. However, *P. pentosaceus* SNF15 annotated xylene degradation, which may have avoided this risk (39, 40). Antimicrobial resistance genes were studied using the CARD, and one antibiotic resistance gene was annotated as penam; cephalosporin belongs to β-lactam antibiotics (41). In our result of the antibiotic resistance experiment, *P. pentosaceus* SNF15 is resistant to ampicillin and tetracycline, which means that although *P. pentosaceus* SNF15 is drug-resistant, it does not directly display at the genome level.

Enterotoxigenic *Escherichia coli* (ETEC) is a common pathogen that induces calf diarrhea (42). Some studies indicated that infection with ETEC can lead to diarrhea, weight loss, organ damage, increased pro-inflammatory cytokines, intestinal permeability, intestinal flora structure disorder, and other pathological changes (43, 44). Taking a variety of probiotics (*Lactobacillus acidophilus*, *Bacillus subtilis,* and *Saccharomyces cerevisiae*) orally contributes to reducing the rate of diarrhea in calves by inducing ETEC K99, promoting calf growth, maintaining the immune system, and intestinal health (12, 45). There is no report about whether *P. pentosaceus* SNF15 can prevent and treat diarrhea and damage in mice infected with ETEC K99. Therefore, we assessed the effects of *P. pentosaceus* SNF15 on mice ETEC K99 infected from clinical symptoms, the intestinal immunologic barrier, the intestinal physical barrier, and the intestinal microbiota structure. In this study, we further revealed that when mice were gavaged with ETEC K99, diarrhea, weight loss, liver atrophy, and infancy occurred compared with the CK group, proving the accomplished establishment of the ETEC K99 infection model. The PPE and PTE groups were gavaged *P. pentosaceus* SNF15 before or after gavaged ETEC K99, respectively. Compared with the CN group, PPE and PTE appear to have decreased fecal scores, body weight recovered, and organ index tended to be expected. This result is similar to Xu et al., *P. pentosaceus* LI05 reduced the mice’s weight loss because of diarrhea (46).

Immunity is a physiological function that resists or prevents the infection of pathogens. The level of inflammatory factors in animal organisms is an essential indicator for assessing immune function (47). ETEC infection increases animal organism IL-6, TNF-α, and IL-1β (48). Oral lactic acid bacteria can reduce the organism’s inflammatory factors, maintaining immune function (49). Our data showed that *E. coli* K99 infection led to mice jejunum levels of IL-6, TNF-α, and IL-1β significantly increaseing. Oral *P. pentosaceus* SNF15 reduces the release of TNF-a, IL-1β, and IL-6 and intestinal inflammatory damage caused by *E. coli* K99 infection. The intestinal barrier is a complex, selective physical barrier that separates and protects the body from the entrance of unwanted microorganisms. Selective permeability is a vital function of the intestinal wall and limits pathogens’ invasion. The impairment of the intestinal barrier leads to elevated intestinal permeability, resulting in the penetration of pathogens that cannot be held back and the infiltration of inflammatory cells, establishing a malignant cycle (49). TJ proteins are one of the intestinal barrier’s basic elements, including occludin, claudin, and the ZO family; their excessive dysregulation leads to immoderate intestinal barrier permeability (51, 52). The mucous layer is a critical constituent part of the intestinal barrier and acts to limit the development of inflammation. MUC2 is a crucial component of the mucous layer released from the goblet cells and has an inseparable relationship with intestinal health and gut microbial structure (53). The investigation revealed that after the *E. coli* K99 infection, the jejunum exhibited severe damage, and the *E. coli* K99 stimulation significantly decreased the expression of MUC-2, occludin, claudin, and ZO-1 in the jejunum mucosa. Intake of *P. pentosaceus* SNF15 could improve *E. coli* K99-infected mice’s intestinal health, significantly increase the content of MUC-2 and TJ proteins in the jejunum, and increase the number of goblet cells in *E. coli* K99-infected mice. Similar to this research, ETEC K88 infection leads to the content of TJ proteins and mucin being significantly reduced in piglets, with the jejunum exhibiting more severe damage (54). Studies indicated that *Pediococcus pentosaceus* IM96 remitting *E. coli* infection leads to intestinal mucosa damage in mice and inflammatory cell infiltration of an increased level of MUC-2 and TJ proteins (55). Thus, we concluded that *P. pentosaceus* SNF15 might protect the integrity of the intestinal barrier by upregulating TJ protein and MUC2 in mice and alleviate the damage caused by *E. coli* K99 infection.

NCD is a common disease of newborn calves, leading to partial calf death. The intestinal microbiome significantly maintains intestinal and general health; a healthy intestinal microbiome can inhibit pathogen colonization in the intestines. Infectious diarrhea is supposed to be the primary cause of intestinal microbiology disorder (55, 56). Receiving antibiotics may result in an imbalance of the gut microorganisms, reducing the abundance and diversity of the gut microorganisms and contributing to colonization and overgrowth of pathogenic organisms (58, 59). A specific diet helps modulate hoetic intestinal microbial composition and activity and maintains the health of the host (40). Studies indicated that the intake of probiotics contributes to intestinal microbial balance and improves or restores the intestinal microbiome (60). Compared with the control group, *E. coli* K99 stimulation of mice and ciprofloxacin oral administration showed a significant decrease in the CHAO1 index, indicating that *E. coli* K99 stimulation seriously affected the richness of the intestinal microbial community of mice and antibiotic treatment did not help to restore the richness of microbial diversity. *Firmicutes*, *Bacteroides*, *Actinobacteria*, and *Proteobacteria* emerged as the dominant microbiota in the infant intestine; imbalance exacerbates the development of diseases (61). The result of the CIP group suggests that oral Ciprofloxacin did not help restore gut microbiome balance. While taking *P. pentosaceus* SNF15 orally prevented these changes. Notably, *Akkermansia* belongs to the phylum *Verrucomicrobia*, mass enrichment in health organisms, involvement in regulating organism intestinal barrier function and immune response, and is supposed to be a probiotic the holds significant potential (62). *P. pentosaceus* SNF15 prevented the decrease of *Akkermansia* due to *E. coli* K99 stimulation, similar to the result by Qiaomai Xu, which is increased abundance of the genus *Akkermansia* was observed in mice treated with *P. pentosaceus* (46). In conclusion, *P. pentosaceus* SNF15 isolated from the feces of healthy calves can effectively alleviate the clinical symptoms such as diarrhea and weight loss caused by *E. coli* K99 infection in mice and regulate the composition of gut microbiology.

## 5 Conclusion

In this study, *P. pentosaceus* SNF15 showed good antibacterial properties against *E. coli* K99, and its antibacterial substances included bacteriocin and hydrogen peroxide. It has good growth and acid production characteristics, is tolerant to bile salt and artificial gastroenteric fluid, has adhesion to Caco-2 cells, is sensitive to broad-spectrum antibiotics, and does not have hemolytic properties. Genome sequencing further revealed that *P. pentosaceus* SNF15 has a strong capacity for carbohydrate metabolism and identified numerous genes related to immunity in terms of anti-oxidant, anti-inflammatory, and antibacterial. In addition, only one antimicrobial resistance gene was predicted in *P. pentosaceus* SNF15 whole genome sequence. In animal tests, we found that taking *P. pentosaceus* SNF15 alleviates the symptoms of *E. coli* K99-induced clinical symptoms in mice, immunological profiles, protects the integrity of the intestinal barrier, improves related pathological features, restores the function of tight junctions and, modulates intestinal microflora composition. Combined, *P. pentosaceus* SNF15 can potentially substitute antibiotics for prevention and treat the calf’s diarrhea caused by *E. coli* K99 infection, but the specific mechanism needs further study.

## Data Availability

The datasets presented in this study can be found in online repositories. The names of the repository/repositories and accession number(s) can be found at: https://www.ncbi.nlm.nih.gov/, PRJNA1069863.

## Footnotes

^1^ https://www.genescloud.cn
