# Corrigendum: Probiotic characteristics and whole genome sequencing of *Pediococcus pentosaceus* SNF15 and its protective effect on mice diarrhea induced by *Escherichia coli* K99

**DOI:** 10.3389/fvets.2025.1593695

**Published:** 2025-04-10

**Authors:** Yalan Su, Mingque Feng, Jingdi Tong, Xiangfu Wen, Meiyi Ren, Deyuan Song, Jinshang Song, Xiaohan Li, Qinna Xie, Jia Cheng, Mingchao Liu

**Affiliations:** College of Veterinary Medicine, Hebei Agricultural University, Baoding, China

**Keywords:** *Pediococcus pentosaceus* SNF15, *Escherichia coli*, diarrhea, probiotic properties, whole genome sequencing, gut microbiota

In the published article, there was an error in Figure 1 as published. Labels C and D are incorrect. The corrected Figure 1 and its caption appear below.

**Figure 1:**
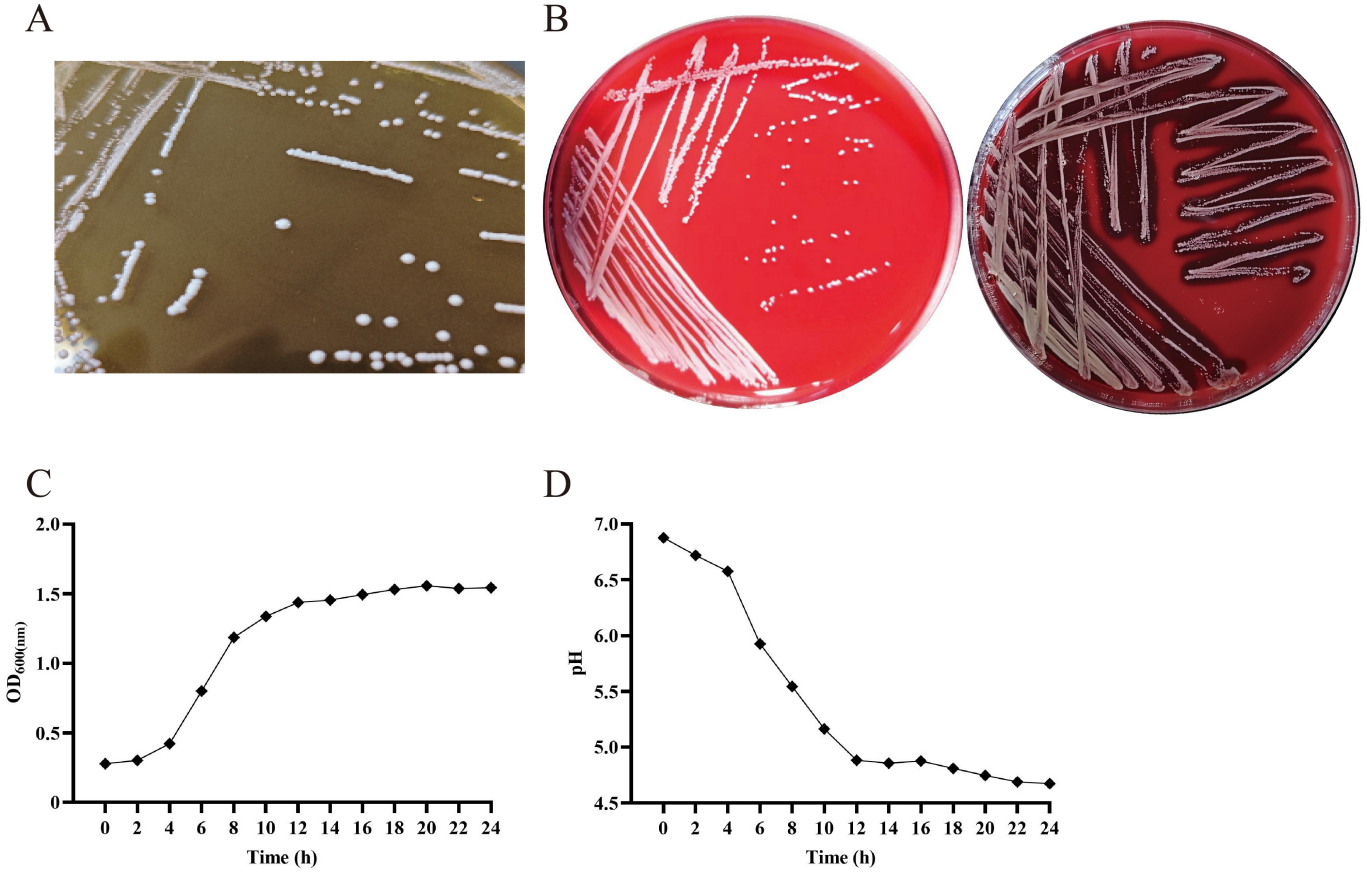
The colony form of *P. pentosaceus* SNF15 is not hemolytic and has good growth performance and acid production ability. **(A)** morphology of *P. pentosaceus* SNF15. **(B)** γ-hemolysis showed *P. pentosaceus* SNF15 **(left)**, β -hemolysis showed by *S. aureus* **(right)**. **(C)** Growth curve analysis of P. pentosaceus SNF15 was performed by measuring OD_600_. **(D)** The acid-producing curve of *P. pentosaceus* SNF15.

In the published article, there was an error in Figure 4 as published. The letters in labels C and E are incorrect. The corrected Figure 4 and its caption appear below.

**Figure 4:**
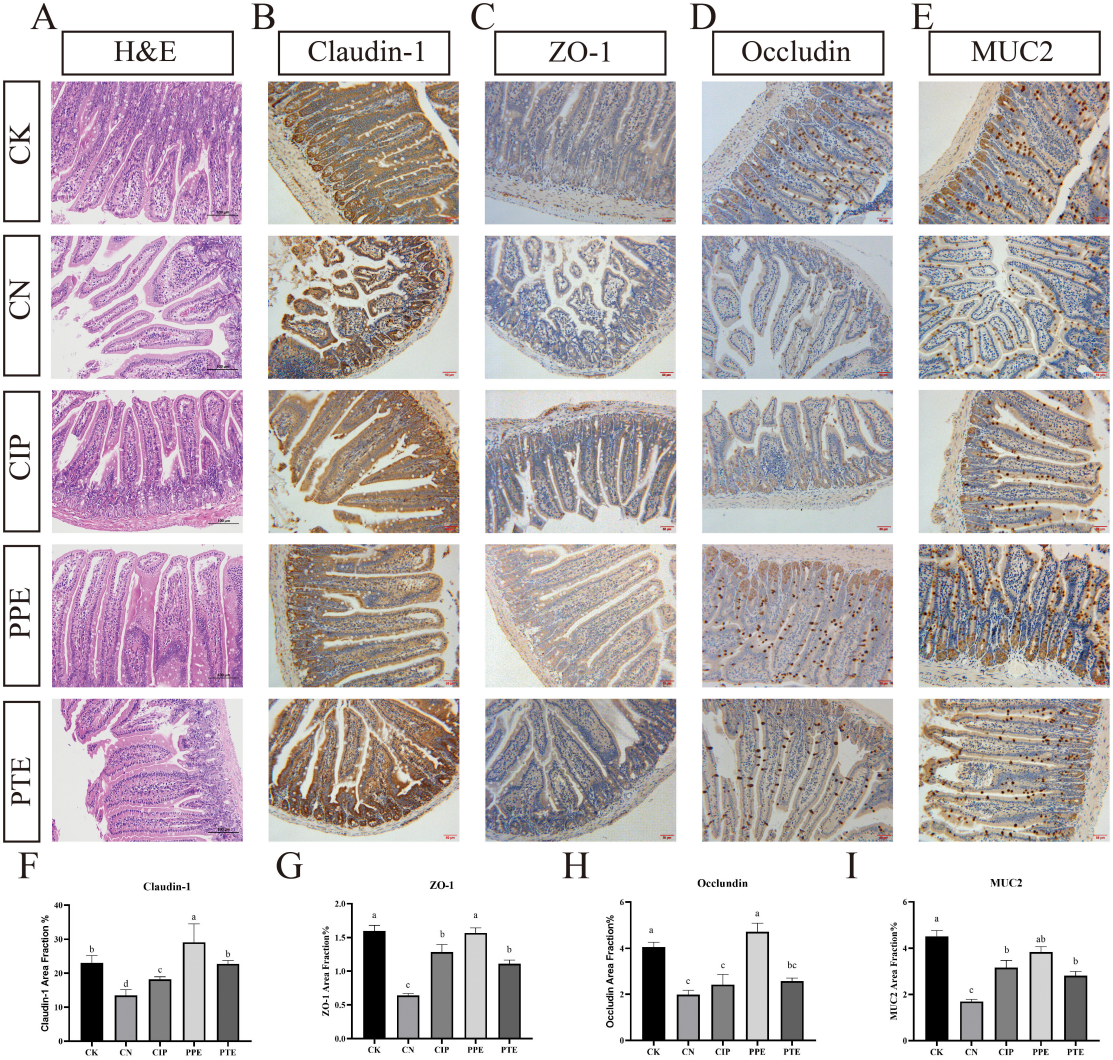
Gavaged *P. pentosaceus* SNF15 and Ciprofloxacin reduced the damage caused by *E. coli* K99 stimulation. **(A)** H&E staining of the jejune tissues. **(B)** Representative immunohistochemical staining of claudin-1 in jejune tissue for each group. **(C)** Representative immunohistochemical staining of ZO-1 in jejune tissue for each group. **(D)** Representative immunohistochemical staining of occludin in jejune tissue for each group. **(E)** Representative immunohistochemical staining of MUC2 in jejune tissue for each group. **(F)** The claudin-1 area fraction for each group **(G)** The ZO-1 area fraction for each group. **(H)** The occludin area fraction for each group **(I)** The MUC2 area fraction for each group. Brown is immunopositive, and blue is immunonegative. The database is expressed as the mean ± SEM. Significant differences were considered at *P* < 0.05. Different letters are marked at bars, without the same superscripts differ significantly (*P* < 0.05).

In the published article, there was an error. The strain culture condition was wrong, “CO_2_” was changed to “O_2_”.

A correction has been made to 2.1. *Isolation, culture, and safety identification of probiotics*, Paragraph Number 1. This sentence previously stated:

The feces were fully homogenized, spread on MRS agar (Qingdao hopebiol, China), and hermetically cultured at 37°C without CO_2_ for 24 h, MRS plate was sealed with a sealing membrane.

The corrected sentence appears below:

The feces were fully homogenized, spread on MRS agar (Qingdao hopebiol, China), and hermetically cultured at 37°C without O_2_ for 24 h, MRS plate was sealed with a sealing membrane.

A correction has been made to 2.1. *Isolation, culture, and safety identification of probiotics*, Paragraph Number 1. This sentence previously stated:

“The purified bacteria were inoculated in MRS broth and static cultured at 37°C without CO_2_ for 24 h, and the centrifugal tube is sealed with a sealing membrane to isolate oxygen.”

The corrected sentence appears below:

“The purified bacteria were inoculated in MRS broth and static cultured at 37°C without O_2_ for 24 h, and the centrifugal tube is sealed with a sealing membrane to isolate oxygen.”

In the published article, there was an error. A unit was misrepresented, and “(1% w/v)” was changed to “(1% v/v)”.

A correction has been made to 2.2.1. *Growth and acid-producing curve curves*, Paragraph Number 1. This sentence previously stated:

“The cultured solutions of isolated strains were added to 80 mL MRS broth (1% w/v) and hermetically incubated at 37°C.”

The corrected sentence appears below:

“The cultured solutions of isolated strains were added to 80 mL MRS broth (1% v/v) and hermetically incubated at 37°C.”

In the published article, there was an error. The incorrect test method, “2.2.1.” was changed to “2.2.2.”.

A correction has been made to 2.2.3. *Bacteriostatic substance*, Paragraph Number 1. This sentence previously stated:

“Use the same way as in 2.2.1 to get *P. pentosaceus* SNF15 supernatant. Catalase or protease K or NaOH (Beijing Solarbio Science & Technology Co., Ltd., China) was added to the supernatant of *P. pentosaceus* SNF15.”

The corrected sentence appears below:

“Use the same way as in 2.2.2. to get *P. pentosaceus* SNF15 supernatant. Catalase or protease K or NaOH (Beijing Solarbio Science & Technology Co., Ltd., China) was added to the supernatant of *P. pentosaceus* SNF15.”

A correction has been made to 2.2.3. *Bacteriostatic substance*, Paragraph Number 1. This sentence previously stated:

“The same method was used as 2.2.1 to determine the inhibitory effect of the treated supernatant on *E. coli* K99.”

The corrected sentence appears below:

“The same method was used as 2.2.2 to determine the inhibitory effect of the treated supernatant on *E. coli* K99.”

In the published article, there was an error. In the experimental group of mice, the experimental treatment of writing errors, “stroke-physiological saline solution” was changed to “*E. coli* K99 (1 × 10^8^ CFU/mL)”.

A correction has been made to 2.4.1. *Animal management and experimental procedures*, Paragraph Number 3. This sentence previously stated:

“(III) CIP group received 200 μL stroke-physiological saline solution at 1-7 days and 200 μL of 50mg/kg Ciprofloxacin solution at 8-14 days.”

The corrected sentence appears below:

“(III) CIP group received 200 μL *E. coli* K99 (1 × 10^8^ CFU/mL) at 1-7 days and 200 μL of 50mg/kg Ciprofloxacin solution at 8-14 days.”

A correction has been made to 2.4.2. *RNA extraction and Quantitative real-time polymerase chain reaction*, Paragraph Number 1. This sentence previously stated:

“qCR assays were performed to assess the relative expression levels of the IL-6, IL-1β, and TNF-α. The expression level of *GAPDH* was used as the reference gene.”

The corrected sentence appears below:

“qPCR assays were performed to assess the relative expression levels of the IL-6, IL-1β, and TNF-α. The expression level of *GAPDH* was used as the reference gene.”

A correction has been made to 2.4.2. *RNA extraction and Quantitative real-time polymerase chain reaction*, Paragraph Number 1. Due to an oversight in writing, the primer sequence was not written. This sentence previously stated:

“All primers were synthesized by Sangon Biotech Co., Ltd. (Shanghai, China).”

The corrected sentence appears below:

“All primers were synthesized by Sangon Biotech Co., Ltd. (Shanghai, China). The specific primers for interleukin were IL-6 (5′-CTTCTTGGGACTGATGCTGGTGAC-3′; 5′-TCTGTTGGGAGTGGTATCCTCTGTG-3′), IL-1β (5′-CCTGGGCTGTCCTGATGAGAG-3′; 5′-TCCACGGGAAAGACACAGGTA-3′), TNF-α (5′-GGACTAGCCAGGAGGGAGAACAG-3′; 5′-CAATGTGTCCGTCGTGGATCT-3′), GAPDH (5′-CAATGTGTCCGTCGTGGATCT-3′; 5′-GTCCTCAGTGTAGCCCAAGATG-3′).”

A correction has been made to 3.2.3. *Adhesion to Caco-2 cells of P. pentosaceus SNF15*, Paragraph Number 1, as the test results were incorrectly described. This sentence previously stated:

“As shown in Table 3, the adhesion rate of *P. pentosaceus* SNF15 on Caco-2 cells is 13.91%.”

The corrected sentence appears below:

“As shown in Table 3, the adhesion rate of *P. pentosaceus* SNF15 on Caco-2 cells is 13.93%.”

A correction has been made to 3.3.1. *Genome assembly result*, Paragraph Number 1, as the test results were incorrectly described. This sentence previously stated:

“It contains 1,855 protein-coding sequences, 1,668,747 bp, accounting for 88.03% of the total length; the average gene length is 899 bp, the max gene length is 8,601 bp, and the min gene length is 90bp. The total repetitive sequence length is 4,435 bp, and the repetitive sequence content is 0.23%. The genome contains 15 rRNA genes and 59 tRNA genes.”

The corrected sentence appears below:

“It contains 1,855 protein-coding sequences, 1,668,747 bp, accounting for 88.02% of the total length; the average gene length is 899 bp, the max gene length is 8,601 bp, and the min gene length is 90 bp. The total repetitive sequence length is 4,435 bp, and the repetitive sequence content is 0.23%. The genome contains 15 rRNA genes and 56 tRNA genes.”

A correction has been made to 3.4.2. *P. pentosaceus* SNF15 attenuated Damages in the mechanical intestinal barrier of *E. coli* K99-infection mice, Paragraph Number 1, as the test results were incorrectly described. This sentence previously stated:

“There was no edema within the lamina propria. Jejunal intestinal villi in the CK group were severely damaged, and there was prominent edema within the lamina propria of the intestinal villi. Compared with the CK group, the intervention of *P. pentosaceus* SNF15 and ciprofloxacin can significantly alleviate the intestinal damage caused by *E. coli* K99.”

The corrected sentence appears below:

“There was no edema within the lamina propria. Jejunal intestinal villi in the CN group were severely damaged, and there was prominent edema within the lamina propria of the intestinal villi. Compared with the CN group, the intervention of *P. pentosaceus* SNF15 and ciprofloxacin can significantly alleviate the intestinal damage caused by *E. coli* K99.”

A correction has been made to 3.4.3. *P. pentosaceus SNF15* attenuated damages in the inflammatory properties' intestinal barrier of *E. coli* K99-infection mice, Paragraph Number 2. This sentence previously stated:

“The CIP group also has the same trends with expression mRNA level, compared with secretion levels of IL-6, IL-1β, and TNF-α significantly decreased with the CK group (*p* < 0.05). The secretion levels of IL-6, IL-1β, and TNF-α in the CIP, PPE, and PTE groups were significantly reduced compared with the CN group (*p* < 0.05) and had no significant difference with the CK group (*p* > 0.05).”

The corrected sentence appears below:

“The secretion levels of IL-6, IL-1β, and TNF-α in the CIP, PPE, and PTE groups were significantly reduced compared with the CN group (*p* < 0.05). The secretion levels of IL-6 and TNF-α in PPE group, secretion levels of IL-1β in CIP group had no significant difference with the CK group (*p* > 0.05).”

The authors apologize for this error and state that this does not change the scientific conclusions of the article in any way. The original article has been updated.

